# An explainable “family bucket” model for simultaneous prediction of K-edge XANES for multiple light transition metals†

**DOI:** 10.1039/d5sc00494b

**Published:** 2025-08-01

**Authors:** Chenyu Huang, Yunjiang Zhang, Shuyuan Li, Huimin Wang, Yaxin Wang, Shihao Wei, Shaorui Sun

**Affiliations:** a Department of Chemical Engineering and Technology, College of Materials Science and Engineering, Beijing University of Technology Beijing 100124 P. R. China sunsr@bjut.edu.cn; b Institute of Matter Science, Beijing University of Technology Beijing 100124 P. R. China

## Abstract

X-ray near-edge structure (XANES) is a crucial bridge between the local structures and chemical properties of materials. Although there have been a number of studies devoted to the development of predictive K-edge XANES spectral models, existing methods are usually still limited to separate modeling for a specific absorbing element. Currently, there is a lack of a K-edge XANES spectra prediction model that can be broadly applied to a wide range of elements, which would enable data dispersed in terms of absorbing elements to be integrated and well utilized. In this work, we develop an innovative “family bucket” model based on a multi-head graph attention convolutional neural network by combining a multi-element mixed dataset with a crystal topology approach for the localized environment. The model is able to predict the K-edge XANES spectra for a wide range of light transition metals (periods 3 and 4) simultaneously. Moreover, it is demonstrated that the training scheme not only improves the accuracy of the model but also the efficiency of its training. In terms of interpretability, several fascinating insights were gained, uncovering the underlying mechanisms of the model for spectral prediction. We investigate the collective behavior of neurons by employing a range of responses to different samples as descriptive features. Notably, the analysis revealed that neurons in the neural network exhibit functional differentiation characteristics analogous to Brodmann areas in the cerebral cortex. The homology of data analysis indicates that the mutual learning of samples from different absorbing elements is occurring between close elements of the same period. Additionally, the attention scores of the samples are determined by both the absorbing element and its surrounding atomic environment. In conclusion, this research advances the understanding of the relationship between XANES spectra and material structures while providing valuable insights into neural networks, enhancing the comprehension of neuronal behavior.

## Abstract 2

An explainable GNN-based “family bucket” model is proposed to simultaneously predict K-edge XANES spectra for multiple light transition metals.

## Graphical abstract

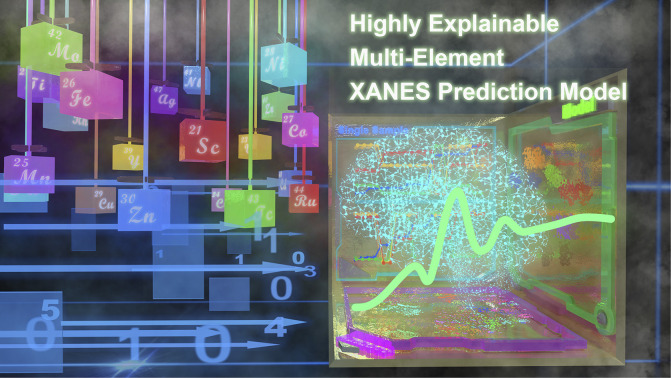

## Introduction

Big data-based artificial intelligence data mining has been called the “fourth paradigm of science”.^1,2^ Compared with first-principles computation based on density functional theory, machine learning has inherent advantages including input liberty, high computational throughput, and short time-consumption.^3^ With the establishment and continuous improvement of a series of large databases for experiments or simulations, such as ChemSpider,^4^ Inorganic Crystal Structure Database (ICSD),^5^ Cambridge Structural Database (CSD),^6^ Materials Project (MP),^7^ Open Quantum Materials Database (OQMD)^8,9^ and Automatic-FLOW (AFLOW),^10^ development in the interdisciplinary field of materials discovery and machine learning is rapidly advancing. Machine learning has been productive in material property prediction,^1,11–13^ assisted characterization,^14–18^ and process optimization.^19,20^ Additionally, there have been many advancements in materials discovery and design by machine learning, as stated by Shi *et al.*^21–28^

In the meantime, X-ray absorption spectroscopy (XAS) has become a popular tool for probing local atomic structure and electronic properties due to its high sensitivity to structural changes and elemental specificity with the development of the fourth-generation synchrotron radiation light source.^29–33^ There is wide interest in analyzing the structure of materials from X-ray absorption near-edge structure (XANES) spectra using machine learning tools. Timoshenko *et al.* decoded information about the average coordination number of metal nanoparticles from XANES using a neural network.^34^ Then, Zheng *et al.* further showed that XANES can predict the oxidation state and coordination number.^35^ At the same time, their work has greatly assisted in the construction of the XANES dataset for simulated computation. Torrisi *et al.* trained and interpreted coordination number, mean nearest-neighbor distance, and Bader charge prediction models with direct intensity information or segmented polynomial fitting information from XANES.^36^ Their research achieves model simplification and functionality extension. The interpretability of their model represents a significant step towards elucidating structural information from XANES spectra.

Compared to the inverse problem, which aims to infer atomic structure information from XANES, there is still less attention paid to the positive problem. Carbone *et al.* developed the first machine learning model for predicting XANES from molecular structures based on a graph neural network.^37^ They trained models that can be used to predict nitrogen and oxygen K-edge XANES for small organic molecules in the QM9 database. Close on the heels of this, Rankine *et al.* published a study on the prediction of Fe K-edge XANES for crystals by using DNN algorithms with RDC as the feature input.^38^ They also used the model to predict XANES for organic systems such as tris(bipyridine)iron(ii). Their results show that there is still a high discrepancy between the crystals and the molecules for the XANES prediction. In addition, it requires an energy-dependent arctangent function for post-processing to obtain better prediction results, which limits the model in some way. In a later study, they replaced the descriptor with wACSF.^39^ Individual models of nine different transition metal elements between Ti and Zn have been created for the prediction of XANES, which demonstrates the versatility of the methodology. However, these models remain isolated between the different elements. Recently, Kwon *et al.* used the DNN algorithm to study the structure and spectrum problem from both positive and negative perspectives in parallel.^40^ For the structure-to-spectrum problem, their study showed that new descriptors such as SOAP achieved great results in the prediction of molecular K-edge XANES, with the best performance for the model using the LMBTR descriptor, which involves angular information, as input. Kotobi *et al.* investigated the interpretability of a series of graph neural network models that predict the small molecule XANES in the QM9 database, based on the work of Carbone's team.^41^ This research is based on the properties of graph neural networks, which greatly breaks the black box of XANES prediction for this kind of model. However, there remains a significant gap in the application of graph neural networks for the prediction of crystal XANES.

Graph neural networks as a machine learning algorithm are popular among chemists for their excellent correspondence between inputs and chemical structures. The graph is a structured data containing points, edges, global information, and an adjacency matrix.^42^ In general, points correspond to atomic codes, edges correspond to bonding information, global information corresponds to the overall properties of a molecule or crystal, and the adjacency matrix records the connections between atoms in chemistry. Through the message passing neural network module, which can usually be divided into three steps of message passing, message aggregation and node updating, the graph neural network realizes the updating of the graph data with an unlimited number of points and edges^43^.^44^ The natural correspondence between graph data and chemical structures, and the ability of graph neural networks to update graphs with an unlimited number of points and edges, has allowed for its frequent application in recent studies.^45–49^

In this study, the prediction of the XANES spectra of crystals with multiple absorbing elements is realized based on a multi-head attention neural network. As far as we know, there is no research report that has developed a graph neural network model that can simultaneously predict crystal XANES spectra for multiple absorbing elements just using the same parameters. In the meantime, Shi *et al.* demonstrated that domain knowledge embedding significantly improved the prediction accuracy of machine learning models in materials property prediction.^50–52^ This study proposes a crystal graph topological method based on a center absorbing atom considering that XANES is a localized atomic structure probe in terms of its physical properties. Benefiting from this topological approach, the data for 20 different transition metal elements were mixed and trained to obtain a more general and accurate multi-element mixed XANES spectra prediction model (MEM-XANES). With the development of various machine learning models, the focus is no longer limited to performance, and the interpretability analysis of models is gradually becoming an integral part in the research of machine learning models.^53,54^ It is worth emphasizing that a novel perspective is introduced here to understand the neural network model. By analyzing the activation patterns and response characteristics of neurons across large-scale samples, revealing their collective behavior, we thereby provide new insights for understanding the complex internal workings of the model. Ultimately, the model is comprehensively analyzed in three dimensions: the neurons of the model, the homology of the data, and the topological structures, with promising results in terms of interpretability analysis.

## Methods

### Workflow

**Fig. 1:**
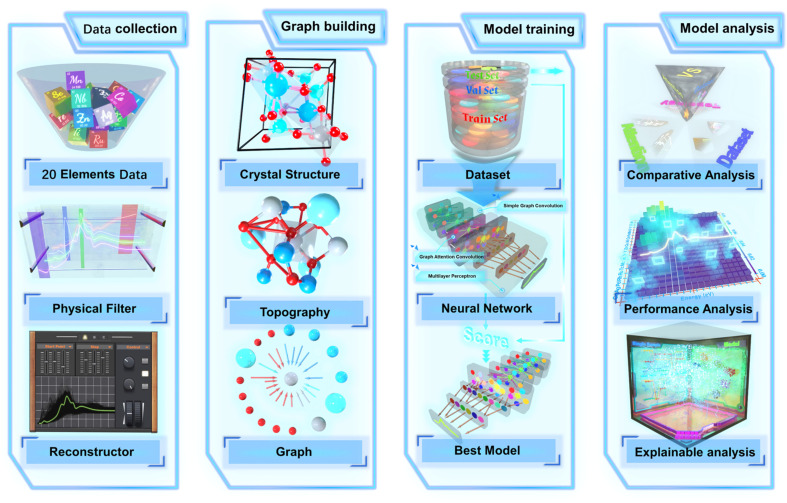
Schematic illustration of the entire multi-element mixed modeling workflow. The workflow is divided into four parts: data collection, graph building, model training and model analysis. Data collection: the spectral data from 20 absorbing elements were collected screened and reconstructed *via* physical filter and reconstructor. Graph building: the crystal structure is topologized and eventually converted to a graph. Model training: the dataset is divided into three parts to iteratively train the network and obtain the best model. Model analysis: a comprehensive analysis of the model in terms of comparative ablation, performance evaluation and interpretability.

The overall flow of this study from data collection and organization to model training and analysis is shown in Fig. 1 from left to right. During the data collection stage, data for 20 different transition metal elements was collected from the original XANES database. The data will be delivered to the filter to screen the spectra by physical common knowledge. The screened spectra will be sent to the reconstructor for alignment and quantitative reconstruction of the spectra from different absorbing elements. The processed data will be topologized from the crystal structure with the absorbing atom as the center and stored as graph data. Before the model training, the graph dataset is divided into three parts (training set, validation set and test set). The training set was directly delivered to the MEM-XANES for training the model by gradient descent. The validation set is used to find the optimal model with the help of the scoring function. Test sets are used to evaluate the model performance and interpretable analyses.

### Dataset

**Fig. 2:**
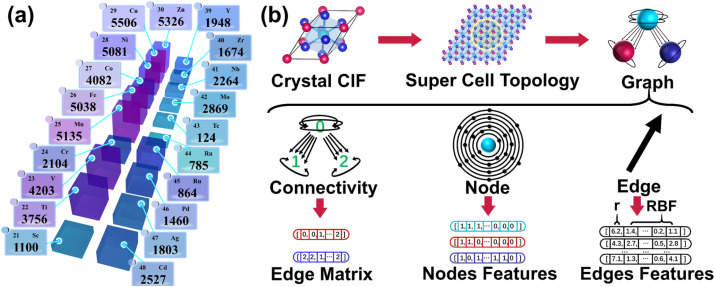
Composition of the dataset and the construction of graph data. (a) The scale of the dataset used in this work. The values indicate the number of samples corresponding to that class of elements in the dataset. (b) The process of constructing graph data is demonstrated from a crystal CIF file. The extension details the three components of graph data, points, edges, and connectivity, along with their corresponding matrix data.

The data is derived from a published dataset from a study on X-ray absorption spectroscopy by Mathew *et al.*^55^ in 2018, which is by far the world's largest library of simulated computed XANES spectra about crystals. K-edge XANES spectra data associated with the 20 transition metal elements from Sc to Zn and Y to Cd were extracted from the library and labeled with a unique ID. For example, an ID of mp-680094-6-Cd-XANES-K means that the data is a Cd K-edge XANES spectrum calculated by constructing a cluster centered on the position 6 atom of the mp-680094 material in the MP database. The ID duplicates and illegal data whose tagged center atom position number exceeds the actual material's atom position number are removed. Afterwards, the four rules were established for filtering XANES data by combining the data screening scheme in the work of Torrisi *et al.*,^36^ which studied spectrum-to-structure based on the Random Forest algorithm. These rules limit the unphysical common-sense cases of negative intensities, excessive peaking, pre-peak stronger than the main peak, and upward trends in the far range. After screening, 57 649 pieces of XANES data were obtained, and the distribution of the data across the elements is shown in Fig. 2a. The mixed dataset for all elements is named EM-Dataset and the subset of a specific element X is called X-Dataset (*e.g.*, Fe-Dataset). Afterwards, the spectra are regularized and reconstructed by X-Dataset. The minimum energy value of the spectra onset in each X-Dataset was counted and an energy window of length 55 eV (20 eV pre-edge region and 30 eV post-edge region, with a 5 eV tolerance) was created using this value as the starting point. The spectra data were reconstructed with 0.5 eV step size to obtain an absorption intensity vector that has a length of 111. The topology crystal structure information makes the raw dataset a graph-structured dataset. These graph-structured datasets were randomly disrupted and divided into training, validation, and test sets in an 8 : 1 : 1 ratio for later research.

### Graph construction

The topological approach of the crystal is shown in Fig. 2b, where an absorbing atom is used as the center to find the nearest 20 neighboring atoms within its 8 Å range after expanding the unit cell for the crystal. If there are less than 20 atoms within 8 Å then virtual atoms with a distance of 8 Å are utilized to fill in the blanks. Atoms are represented by the points, and the connectivity between atoms is represented by the adjacency matrix to form a directed graph. The 118-dimensional electron arrangement is used to encode the atom for the task to predict XANES against. The edge information is jointly represented by the distance between atoms and a 64-dimensional vector of distance unfolding *via* a Gaussian radial basis function so that the neural network can autonomously select effective features.^45^ The Gaussian radial basis function is specified as follows:

1 RBF = *ϕ*(*r*_*ij*_)·exp(−*β*_k_(exp(−*r*_*ij*_) − *μ*_k_)^2^)

where *μ*_k_ and *β*_k_ are the center and width of the Gaussian function, respectively. *ϕ*(*r*_*ij*_) is the smooth cutoff function, which is formulated as follows:2
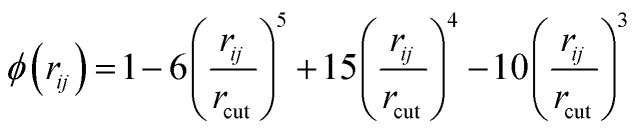


### Graph neural network

**Fig. 3:**
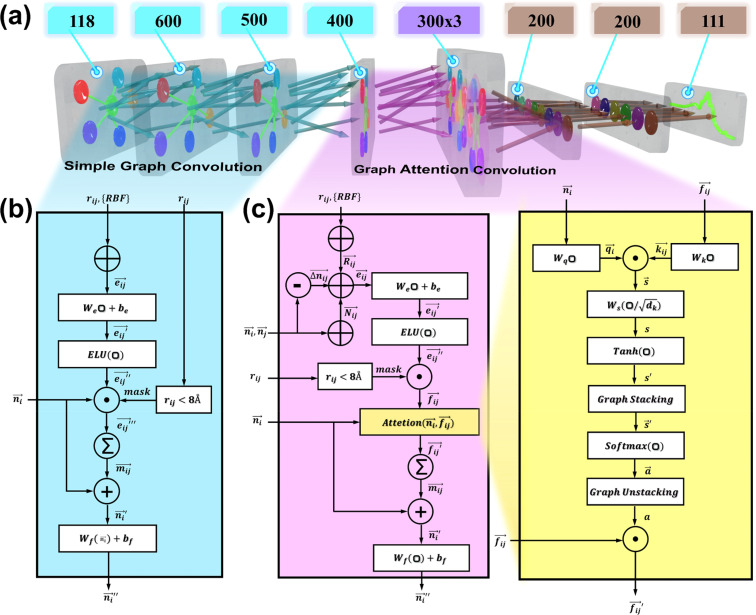
Overview of the MEM-XANES model architecture. (a) Schematic of the connection sequence and the composition of the modules in the network. (b) Flowchart of the simple graph convolution computation. (c) Flowchart of the graph convolution computation with attention mechanism. Compared to the simple graph convolution, the graph convolution with attention mechanism automatically selects the complicated input features by calculating the attention scores.

Network architecture. As shown in Fig. 3a, the network architecture of this study can be divided into seven layers, and the number of features from the input to the output nodes is [118, 600, 500, 400, 300 × 3, 200, 200, 111] sequentially. The 118-dimensional node features in the graph data are transformed into 400-dimensional node features after three simple graph convolutional processes. The transformed graph data was copied to three graph attention heads separately to extract the XANES data. The graph data processed by the multi-head graph attention convolutional layer will be pooled into 900-dimensional features and downscaled into 200-dimensional features by dense layers. Finally, it is transformed to 111-dimensional X-ray absorbed intensity vectors corresponding to the energy by two linear layers. The computation flows for the simple graph convolution and graph attention convolution are shown in Fig. 3.

## Results & discussion

### Dataset

**Fig. 4:**
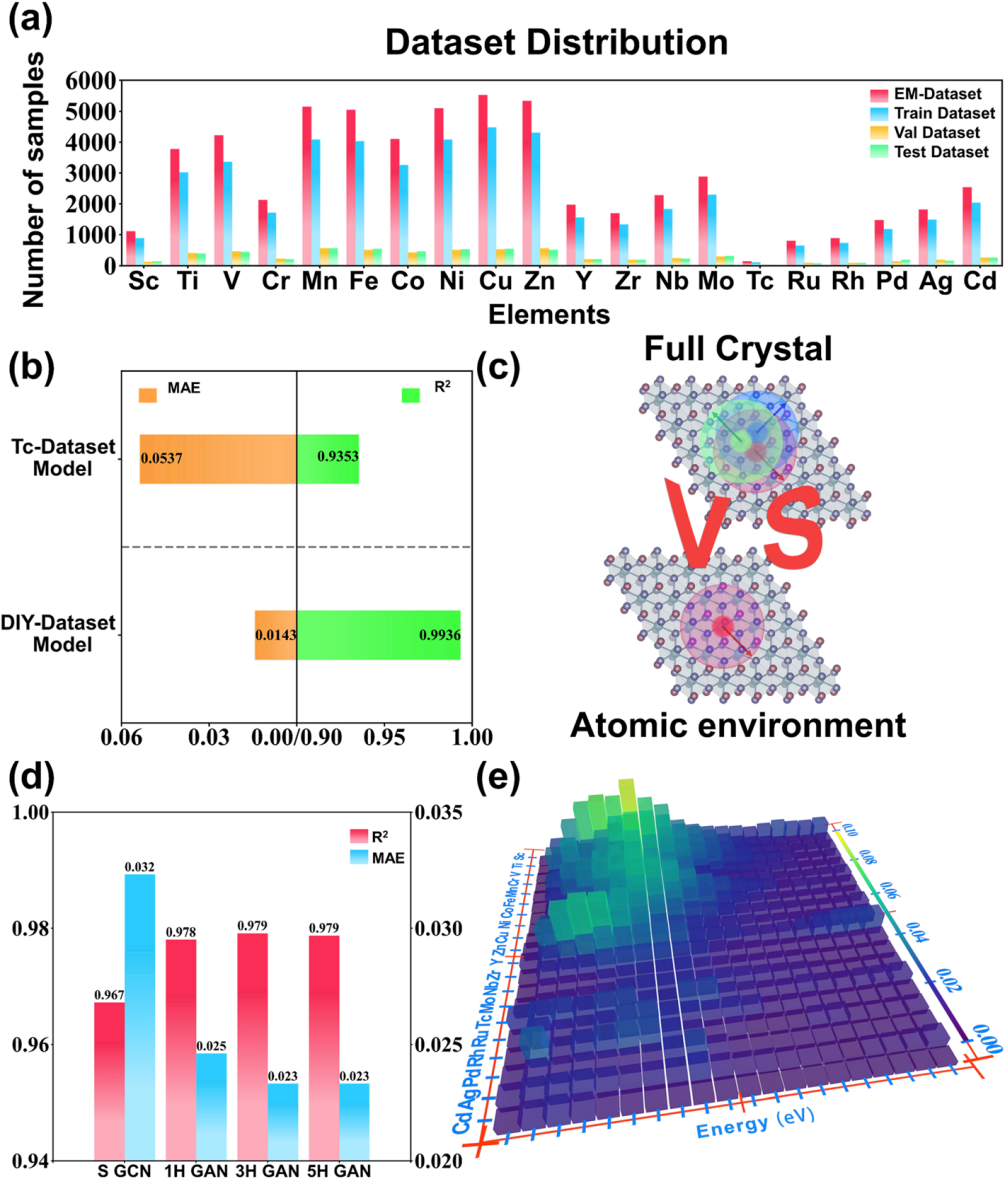
Evaluation of model performance. (a) Distribution of samples among different elements after dividing the dataset. (b) The comparison of MAE (orange) and *R*^2^ (green) performance evaluation on the same Tc-element test set for the models trained by a single Tc-element dataset and a multi-element mixed dataset. (c) Schematic of the comparison of two topological approaches: the regular full crystal topology *versus* the targeting localized atomic environment topology. (d) Graph attention convolution module gradually ablates (S GCN: simple graph convolution; *x*H GAN: *x*-head graph attention convolution) *R*^2^ (red) and MAE (blue) performances. (e) Distribution of mean MAE for different elemental samples after binning by energy.

For exploring the effect of elemental mixed dataset in training model for this work, different datasets were replaced for training and scoring comparisons. First, the scores of the 20 X-Dataset trained models were compared to the EM-Dataset trained model by weighted average of the sample proportions. To ensure the fairness of the two comparisons, ideally, the distribution of elements in the training, validation and test sets divided by the EM-Dataset should be the same as the EM-Dataset. Visualizing the distribution of elements before and after the EM-Dataset division, their distributions are indeed very similar, as shown in Fig. 4a. The weighted score of the models trained on each of the 20 X-Datasets is compared to the score of the model trained on EM-Dataset as shown in Table 1. For stability, the scores presented in Table 1 are averaged over multiple runs and the detailed single run results can be found in Table S2.† The mean absolute error (MAE) of the EM-Dataset trained model is 0.0233, which is decreased compared to the weighted average MAE of 0.0269 for the X-Dataset trained model. The *R*-squared (*R*^2^) of the EM-Dataset trained model is also improved in comparing both, which suggests that the different elemental datasets play a role in helping each other. At the same time, a special case exists in the dataset. The number of samples for the Tc element was only 124, which created an opportunity for research. As we all know, it is difficult to obtain an excellent result from training on a complex model like a neural network for a small dataset such as the Tc-Dataset. The EM-Dataset with the Tc-Dataset removed was mixed with the training and validation sets divided from the Tc-Dataset as a customized dataset (DIY-Dataset). Then, the DIY-Dataset was redivided into a training set and a validation set in a ratio of 9 : 1. Finally, the test set that was divided from Tc-Dataset was kept as a uniform test set to evaluate the DIY-Dataset trained model and the Tc-Dataset trained model. The result is shown in Fig. 4b, where the model trained on the DIY-Dataset scores surprisingly well with MAE and *R*^2^ of 0.0143 and 0.9936, respectively. The result further demonstrates the superiority of the EM-Dataset, which was used as the default dataset in subsequent studies.

**Table 1:** Performance comparison of two training strategies

	EM-Dataset	Weight-X-Dataset
MAE	0.0233	0.0269
*R* ^2^	0.9791	0.9738

### Topological approach

A regular way to topologize a crystal is to topologize each atomic environment in the CIF file and finally merge them into graph data, as shown in Fig. 4c. For the physical properties of XANES that are sensitive to the local atomic environment, a topological approach based on the centered absorbing atom has been dedicated in this study. Foreseeably, this atomic environment topology approach will surely reduce the computation time. Both topological approaches were used to train the model on the EM-Dataset considering the specific impact of the two topological approaches. In fact, the performance of the model trained by utilizing the full crystal topology approach to composition as shown in Table 2 has an MAE of 0.0470 and an *R*^2^ of 0.9149, which is far inferior to the atomic environment topology. This disparity is caused by the fact that the full crystal topology approach makes it difficult for the neural network to pin down absorbing atoms or absorbing elements on the EM-Dataset. In addition, unsurprisingly, the time it takes to train the model on graph data is greatly reduced by the topological approach based on the atomic environment of the absorbing atom. The full crystal topology graph data takes about 7.17 times longer to train than the atomic environment topology. Therefore, the new topological approach not only improves the training efficiency but also improves the prediction accuracy of the model. At the same time, this is the reason why the model was able to be trained on the EM-Dataset.

**Table 2:** Performance comparison between two topologies

	Full crystal topology model	Atomic environment topology model
MAE	0.0470	0.0233
*R* ^2^	0.9149	0.9791
Time per epoch (s)	57.6210	8.0402
Total time (h)	12.8047	1.7867

### Model ablation

Ablation analysis experiments were conducted to investigate whether and how much the multi-head graph attention mechanism plays a role. Models were trained using simple graph convolution, single-head graph attention convolution, three-head graph attention convolution, and five-head graph attention convolution as the last convolutional layer (layer 4) of the network. The model's scores are shown in Fig. 4d, which shows similar results for the MAE and *R*^2^. There is a significant difference between the model using simple graph convolution compared to the model using graph attention convolution, with about a 0.01 difference in MAE. The operation from single to multiple heads also improves the performance of the model. However, both scoring functions remained essentially unchanged when the number of heads was increased from three to five. This suggests that constantly increasing the number of heads is meaningless. In other words, crudely increasing the model parameters does not improve the model performance, which illustrates the role of the graph attention mechanism in another direction. Therefore, the three-head graph attention convolution model is treated as the final model.

### Performance presentation

**Fig. 5:**
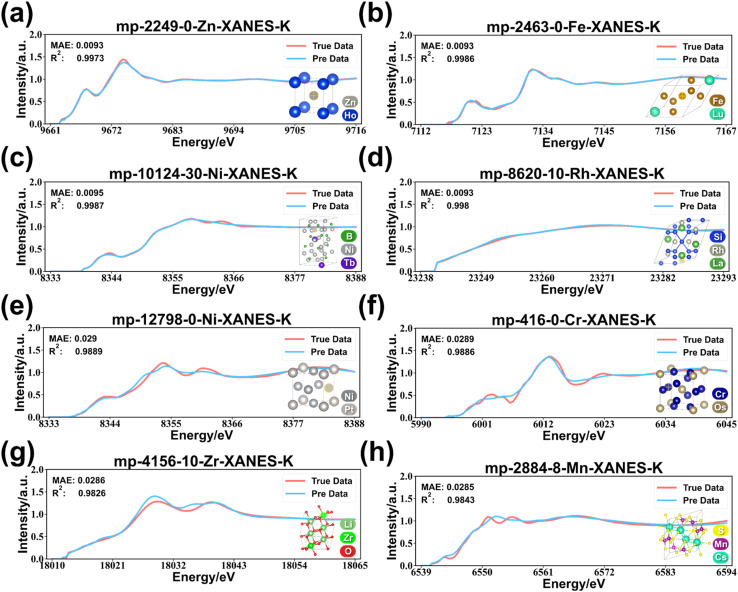
Prediction samples. (a)–(d) Comparison between the calculated and predicted values for samples in the first quartile (Q1) sorted by MAE from low to high. (e)–(h) Comparison between the calculated and predicted values for samples in the third quartile (Q3) sorted by MAE from low to high.

The MAE and *R*^2^ of the best model are shown in Fig. 4d, which are 0.0233 and 0.9791, respectively. To insight into the predictive ability of the model, the absorption intensities predicted by the model in the test set were binned by energies and elements to calculate the MAE with the true values yielding the thermograms shown in Fig. 4e. The error range indicates that the error between the true value and the predicted value is from 0.00 to 0.10, and is not simply clustered around the mean of 0.02. In terms of energy, the model generally predicts more accurately in the high energy region, in which the MAE is generally below 0.03. In terms of elements, the model predicts XANES more accurately for the 5th period elements with MAE below 0.03. For the 4th period elements, the overall MAE of the model is below 0.06, but there are regions of energies where the MAE exceeds 0.06 for the elements V and Sc. In particular, the model even has regions with MAE of 0.09 when predicting K-edge XANES for Sc element. However, what is presented in Fig. 4e is still the result after averaging. To further observe the prediction of XANES, Fig. 5 presents the four samples at the 25% and 75% quantiles following the sorting of MAE from low to high (more in Fig. S1 and S2†). As shown in Fig. 5a–d, the predicted XANES for samples with MAEs at 25% basically overlap with the true XANES, and the model reproduces almost all peaks for these samples. There are slight disparities between the predicted and true values in some of the details, and the MAE is around 0.0094. The predicted XANES for samples lying at 75% can still overlap with the true XANES in terms of the overall trend, and the MAE is usually about 0.0289. The positions of the main peaks were generally predicted accurately, with larger deviations in the absorption intensities. In fact, the predictions for these samples lose more detail as shown in Fig. 5e–g. For example, the prediction for sample mp-416-0-Cr-XANES-K is not sufficiently accurate in reproducing the fine features of the absorption intensity, particularly at the pre-edge peak and in the region after the main peak. As a result, the model is able to predict K-edge XANES for a variety of elements simultaneously and retains an acceptable accuracy for about 75% of the data. However, the average prediction accuracy for Sc and V still needs to improve.

### Interpretability analysis

Interpretability analysis is an important part of the process of evaluating a model.^56–59^ It increases trust for a model while also allowing us to notice flaws and bugs in the model. In the next section, our MEM-XANES model will be analyzed from three dimensions: the neurons of the model, the homology of the data, and the topological structures.

### Interpretability of the neurons of the model

**Fig. 6:**
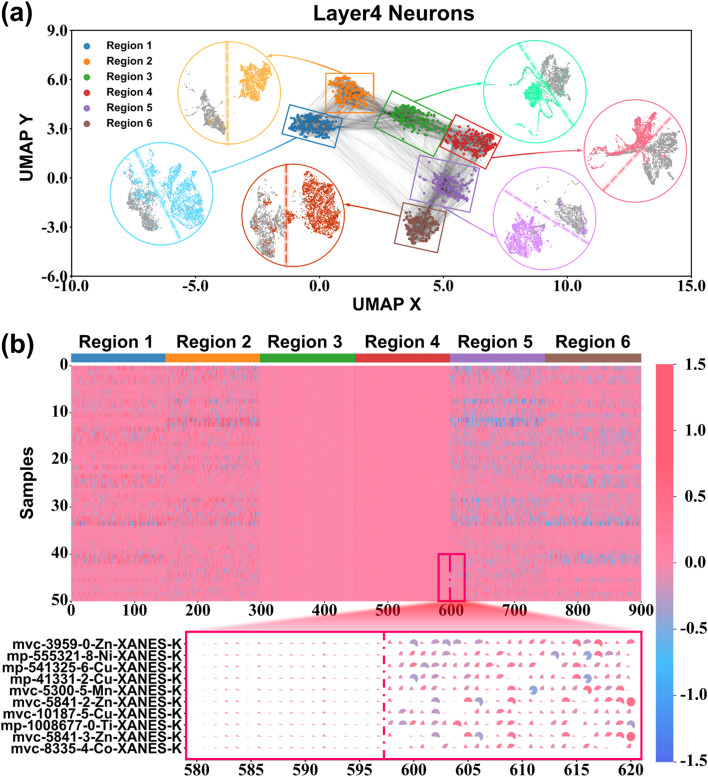
Visualization of the behavior of the model neurons. (a) The result after coloring the neurons in layer 4. The inset illustrates the result of binary classification by a specific rule based on features provided by a set of neurons for samples after UMAP dimensionality reduction. (b) Response dynamics of layer 4 neurons for a set of samples. The inset is a localized zoomed-in result of the response dynamics.

To break the black box of deep learning, a new perspective is introduced in which six layers of neurons in the network, excluding the input and output layers, are analyzed by the UMAP. The behavior of the neurons is characterized by response dynamics, which are the activation values produced by neurons when processing a series of samples, collected from all the samples in the test set. An extremely striking result is that the neurons in layer 4, unlike the other layers, are clearly clustered into six groups (as shown in Fig. S3†). To further investigate the significance of each group for the model, the six neuronal groups were manually divided and stained, and the results are shown in Fig. S4.† After that, the sample features are analyzed by dimensionality reduction, which is computed from these six different neuron groups. As shown in Fig. 6a, there is a unique rule for each group of neurons that distinguishes the samples into two categories, which reveals the differentiated “thought patterns” between different groups of neurons. It is remarkable that there is functional differentiation among neurons, akin to the specialization of different regions in the human cerebral cortex for processing different information.

The rule for region 1 is whether the average first ionization energy of the near-neighboring atoms is greater than 850 kJ mol^−1^. The region 2 rule is whether the two nearest neighboring atoms are N, O or F. This may be due to the consideration that highly electronegative near-neighboring atoms have a greater influence on the electron cloud of the central absorbing metal atom.^60–62^ Regions 3 and 4 are both divided relying on the first shell layer (Δ*r* < 0.6 Å). Region 3 is divided according to whether the average first ionization energy of the first shell layer is less than 1300 kJ mol^−1^. Region 4 is ruled by whether the average distance of the first shell layer is less than 2.24 Å. In fact, the first shell layer is exactly the concept commonly used in X-ray near-edge absorption spectroscopy.^63–66^ The rule for region 5 is whether the nearest-neighbor atom distance is less than 2.20 Å. This is similar to region 2, which also considers the nearest-neighbor atoms. The rule for region 6 is whether the average distance of neighboring atoms within 2.70 Å is less than 2.30 Å. The results for the source attribution of neurons (Tables S6–S11†) suggest that the clustering of these neurons and the functional results are not coincidental. Basically, each attention head corresponds to two neuron clusters and there are similarities in the rules between homologous neuron clusters. For example, almost all of the neurons in regions 1 and 6 come from the first attention head, while both rules take into account the average information of the broad near-neighbor atoms. Regions 2 and 5 from the third head are more focused on the 1–2 nearest-neighbor atoms. Regions 3 and 4 are both considered from the first shell layer. As a matter of fact, this is consistent with the UMAP result for neurons, which corresponds to the many gray lines connecting area 1 and area 6, area 2 and area 5, and area 3 and area 4.

In addition, the specific response of neurons to a cluster of samples is shown in Fig. 6c. It is obvious that the response dynamics of neurons in regions 3 and 4 to different samples are different from those in the other regions. The local zoom results further indicate that neurons in regions 3 and 4 respond with very weak dynamics for this cluster samples compared to the other regions. This almost inactivated state is similar to recent findings in human neuroscience.^67^ Thus, the neurons of the MEM-XANES model have behaviors like a human brain whose different functional regions focus on processing different information. The MEM-XANES model analyzes the local structure of the crystals to obtain the X-ray near-side absorption spectra from the nearest atoms, the first shell layer, and the broad near-neighbor atoms. The phenomenon of different regions being responsible for processing distinct information is quite similar to the specialized processing mechanisms observed in the Brodmann areas of the cerebral cortex. Meanwhile, the comparison of the activated and inactivated states of neurons in different regions when confronted with a cluster of similar samples further corroborates this notion. Additionally, the normative thinking of neurons and the interpretability of their behavior may require the embedding or guidance of innate computational mechanisms. Attention mechanism is one of the powerful tools that can help the neural network brain to evolve.

### Interpretability of the homology of data

**Fig. 7:**
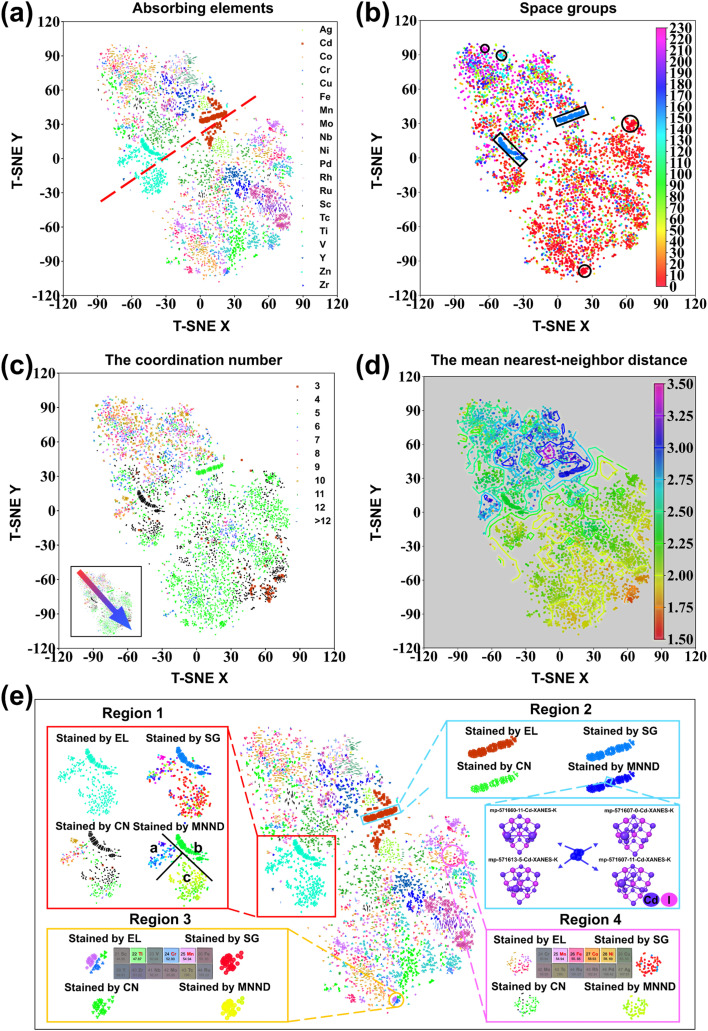
Visualization of samples reduction on the test dataset. (a) Absorbing elements (EL) stained map for sample reduction. (b) Space group (SG) stained map for sample reduction. (c) Coordination number (CN) stained map for sample reduction. (d) Mean near-neighbor distance (MNND) stained map for sample reduction. (e) Multi-stained contrast map based on absorbing elements map. Regions 1 and 2 are the dispersed and dense regions for the samples with the same absorbing element, respectively. The inset of region 2 is the topology of the corresponding samples in a localized zoom of region 2. Regions 3 and 4 are the dispersed and dense regions for the samples with different absorbing elements, respectively.

For intuitive comprehension of the role of graph convolution in encoding crystal information at the dataset level, the test set was projected into two dimensions and analyzed by T-SNE technology. Specifically, T-SNE dimensionality reduction is applied to the last graph convolutional layer output, which is a 900-dimensional graph data encoding. Staining visualization of the reduction results based on absorbing elements, space groups, coordination numbers, and mean nearest-neighbor distance is shown in Fig. 7a–d. As shown by the absorbing element staining maps in Fig. 7a, samples of the same absorbing element tend to cluster, most notably for the elements Cd and Zn, with the two species essentially clustered into two groups. This confirms that the MEM-XANES model has the ability to distinguish between samples of different absorbing elements. However, it is important to note that the samples, which have the same absorbing elements, may also be multi-clustered or even dispersed distributionally. In total, this dispersion is bounded by the red dotted line, showing that samples of the same element are distributed both above and below it. This phenomenon insightfully reveals the inherent complexity in embedding space. The distribution of samples in the embedding space is not only determined by absorbing elements, but also influenced by other hidden laws. For space group staining, the regularity is not significant, showing a few sporadic clusters, which are framed by a solid black line in Fig. 7b. It was due to the fact that the processing during the graph data construction stage largely lost the information about the crystal structure in terms of space. A clearly patterned distribution is then observed in the coordination number staining maps, as shown in the inset of Fig. 7c, where the coordination number shows a decreasing trend from top left to bottom right in the embedding space. For the mean nearest-neighbor distance a continuous variation can be observed, as shown in Fig. 7d. This continuous variation even allows us to sketch the corresponding contours.

To further investigate the properties of the embedding space, the absorbing element-stained map was used as a base map for detailed comparative analysis with other stained maps. As shown in Fig. 7e, region 1 is an oversized cluster where only Zn absorbing element samples are clustered. The samples from this cluster can be further divided into subregions *a* (>2.75), *b* (≈2.50), and *c* (<2.25) based on the mean nearest-neighbor distance. Meanwhile, the space groups in region *b* are nearly identical, and the coordination number of samples in region *a* is essentially more than 5. It suggests that the complexity of the sample embedding space is caused by a multiple combination of different information. The dispersion of absorbing elements bounded by the red dashed line in Fig. 7a is explained. For region 2, there is a tightly clustered group of Cd absorbing elemental samples. It is difficult to distinguish between them for all four staining maps: absorbing elements, space groups, coordination numbers, and mean nearest-neighbor distance. To explore such a situation, the samples were further analyzed by drawing the topologies that corresponded to a smaller region of the samples. It is not difficult to find that the topologies of these samples from different crystals (Fig. S5†) or different local structures of the same crystal are very similar. They only differ in the two outermost atoms and in the interatomic distances. This makes perfect sense, just as humans categorize similar situations while still retaining the distinction between them. Region 3 is a dense, small cluster where samples of multiple absorbing elements are present. In addition to their essentially identical space groups, coordination numbers, and mean nearest-neighbor distance properties, there is a contribution of absorbing elements for their clustering together. The elements Ti, Cr and Mn just happen to be close to each other in the same period. It is not unique, and similar to the case in region 4, where the sample was relatively dispersed with differences essentially only between absorbing elements. Mn, Fe, Co, and Ni are just neighbors in the same period. Again, it provides convincing evidence that the MEM-XANES model can enable mutual learning between samples of different elements. It is also further revealed that such mutual learning is accomplished by analogizing similar samples of closely absorbing elements in the same period. In summary, the local information of the crystal is transformed to a multi-dimensional, semantic divisible space by graph convolutional encoding. Besides the space groups, the absorbing elements are the core of the MEM-XANES model, while the coordination number and the mean nearest-neighbor distance have been proved to be important bridges between XANES and the structure by Torrisi *et al.*^36^ Consequently, the MEM-XANES model in this study does accomplish the task of encoding the crystal structure information into multi-dimensional vectors that are highly correlated with the XANES. Moreover, it also provides a further explanation for the multi-element mixture model to realize mutual learning among samples of different elements.

### Interpretability of the topological structures

The attention mechanism is not only a useful tool to improve the performance of the network, but its attention scores are also an important bridge between the process of networks processing formalized data and human cognition, which is of great significance for enhancing the interpretability of the model.^68–70^ Therefore, the attention scores are utilized for interpretability analysis of the topological structures. Samples with high prediction accuracy were assumed to have higher interpretability of results, and 10 samples with different absorbing elements were randomly selected from a subset with the top 1000 MAE rankings of the test set for analysis. Specifically, maximum–minimum standardization was applied to the attention scores of each of the three heads from the sample, with which the differences between the attention scores were analyzed (Table S12–S21†). The analysis revealed that the samples can be divided into two cases based on the presence or absence of mathematically equivalent atoms of the central absorbing atom in the group of near-neighbor atoms. Sample mp-1005986-26-Mo-XANES-K (absence) and sample mp-640381-6-Cu-XANES-K (presence) are illustrated as examples here. It is necessary to state that other samples show the same rule.

**Fig. 8:**
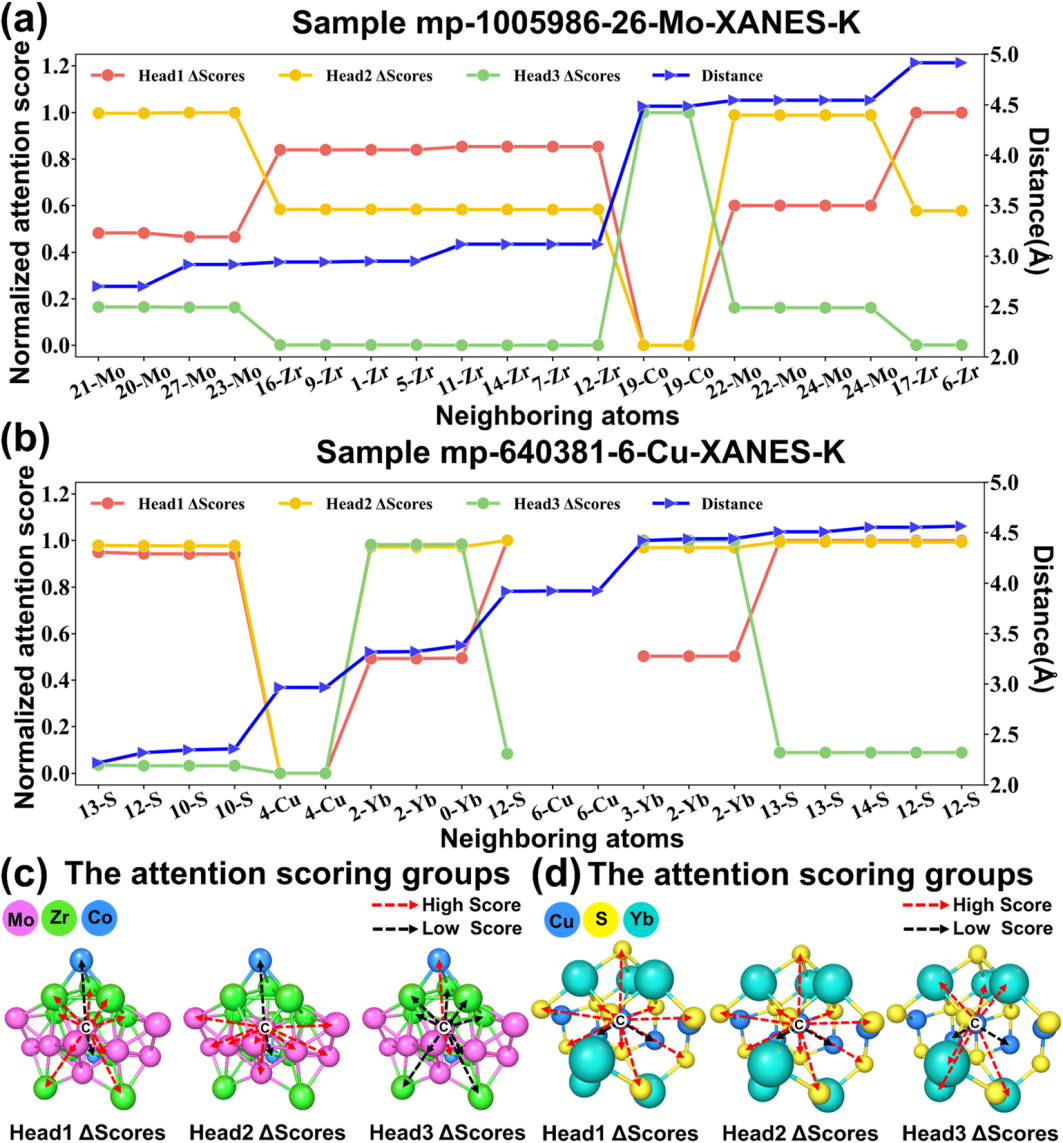
Visualization of sample attention scores. (a) The distribution of near-neighbor atoms for sample mp-1005986-26-Mo-XANES-K with the corresponding attention scores for the three heads. (b) The distribution of near-neighbor atoms for sample mp-640381-6-Cu-XANES-K with the corresponding attention scores for the three heads. (c) The attention high/low scoring element group labels for the three heads for sample mp-1005986-26-Mo-XANES-K. (d) The attention high/low scoring element group labels for the three heads for sample mp-640381-6-Cu-XANES-K.

By arranging the near-neighbor atoms in order of proximity, the attention scores from three heads for the sample mp-1005986-26-Mo-XANES-K are shown in Fig. 8a. For a single attention head, the attention scores for the same element are concentrated in the same zone, while the zones of different elements do not overlap. For example, in the first head, all Zr atoms have relative scores greater than 0.8, Mo atoms have scores between 0.7 and 0.4, and Co atoms have scores less than 0.1. The attention scores for the three heads are similar, which demonstrates that the MEM-XANES model's attention mechanism is discriminatory for elemental species of near-neighbor atoms. It is interesting to mention that the attention scores of the first head also exhibit difference between neighboring near-neighbor atoms of the same element. Specifically, the second nearest-neighbor atom (20-Mo) and the third one (27-Mo) were significantly different on the first head's attention scores. It is not difficult to observe that this difference is caused by the change in distance, rather than the difference in nodes, by comparing the distance line between the near-neighbor atoms and the central atom. As Table S12† shows, this situation is also present in the other two heads, but the first head is more sensitive to the change in distance. Further, the highest scores element group and the lowest scores element group of the different heads are marked on the topology, and the results are shown in Fig. 8c. In similar fashion to recent findings, the model's attention scores also showed complementary and synergistic behavior across the different heads.^71–73^ For sample mp-640381-6-Cu-XANES-K (shown in Table S17†), when the graph equivalent atoms of the center absorbing atom are present in the near-neighbor atoms, these atoms receive high attention in all three heads. It is not difficult to understand that the center absorbing atom is certainly the most important part of the whole system. After excluding such atoms, the normalized results and the high/low scoring elemental group labeled results are shown in Fig. 8b and d, respectively, which are like the samples with no central absorbing atoms.

### Calculated validation

**Fig. 9:**
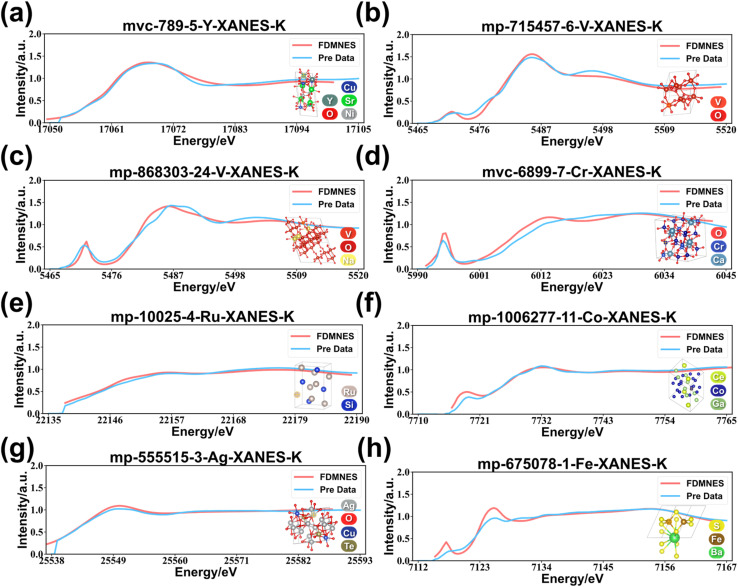
Validation of FDMNES simulations. (a)–(h) Comprehensive comparison of XANES between model predictions and FDMNES simulations for samples.

To rigorously evaluate the physical reliability of the model, high precision FDMNES^74–76^ benchmark simulations were performed on 24 representative samples (Fig. 9 and S6†) spanning diverse elements, coordination numbers, oxidation states, and local symmetries. To focus the comparison on the physically crucial spectral shape, all spectra were energy-shifted and intensity-normalized, correcting for the inherent systematic discrepancies in absolute energy and absorption cross section calculations between theoretical methods.^77,78^ The results indicate that beyond excellent agreement in overall spectral shape, the model has crucially captured the transition selection rules governed by local symmetry.

As shown in Fig. 9a, the first coordination shell of the central Y atom in sample mvc-789-5-Y-XANES-K is composed of six oxygen atoms with Y–O bond lengths of 2.36, 2.36, 2.40, 2.40, 2.42, and 2.42 Å, forming a highly symmetric, nearly centrosymmetric octahedral local environment. According to the selection rules, this high degree of symmetry strongly suppresses the dipole-forbidden 1s → 4d electronic transition. Thus, the nearly perfect overlap of the two curves at the smooth absorption edge, which is dominated by the dipole-allowed 1s → 5p transition, confirms the physical reliability of the model. In stark contrast, a sample also featuring a six-coordinate oxygen environment around a central V atom exhibits a distorted octahedral geometry, with V–O bond lengths distributed unevenly over a wider range (1.91–2.20 Å). This configuration breaks the central inversion symmetry. In such a non-centrosymmetric crystal field, the 3d orbitals of the central V atom hybridize with the 2p orbitals of the surrounding oxygen atoms. This p–d hybridization lends partial p-character to the otherwise pure d-orbitals, thereby opening the formerly dipole-forbidden 1s → 3d electronic transition channel. As shown in Fig. 9b, the resulting pre-edge peak at ∼5472 eV serves as a direct spectroscopic fingerprint of this distorted octahedral environment. The model's success in predicting the presence, position, and general intensity of this key feature provides strong evidence that it has learned the core quantum-mechanical causal chain from structural symmetry breaking to the emergence of specific spectral features. Furthermore, another sample (Fig. 9c), also a V-centered distorted octahedron, presents an even more extreme case. With a bond length distribution spanning 0.77 Å, featuring a very short V=O double bond (1.63 Å) and a very long, weak V–O bond (2.40 Å), its local environment deviates severely from centrosymmetry. This extreme distortion induces a much stronger p–d hybridization, which boosts the transition probability and consequently yields a pre-edge peak of greater intensity. The direct comparison of these two distorted vanadium octahedra reveals a remarkable capability in that the model not only predicts the existence of the pre-edge peak but also quantitatively captures the crucial difference in their intensities. This further confirms that the model has developed a profound understanding of the physical laws connecting local symmetry to electronic transition channels. Fig. 9d showcases a tetrahedral coordination environment, a geometry that inherently lacks a center of inversion. This leads to a highly allowed 1s → 3d transition, corresponding to the extremely intense pre-edge peak observed in the XANES spectrum at ∼5993 eV. This progression, which ranges from predicting no pre-edge peak for a nearly perfect octahedron to a weak peak for a distorted one, a stronger peak for a more extremely distorted one, and finally a very intense peak for the tetrahedral environment, provides irrefutable evidence of the model's physical reliability.

Moreover, the model demonstrated robust physical generalization capability across systems spanning different chemical bonding types and electronic structure constraints. In intermetallic compounds (Fig. 9e and f), the model correctly predicts the smooth edges or shoulder-like features dominated by delocalized bands, rather than localized pre-edge peaks. For the Ag system, where the 4d orbitals are fully occupied and thus there is no 1s → 4d transition channel (Fig. 9g), the model also correctly predicts the absence of a pre-edge peak. Together, these successful validations depict a reliable model that has grasped core physical principles. At the same time, the presence of quantitative discrepancies in the sulfide system (Fig. 9h) transparently indicates current model limitations and directions for future optimization, demonstrating that the assessment of model capabilities is both objective and comprehensive.

## Conclusion

In summary, this work introduces a multi-head graph attention convolutional neural network for crystal XANES prediction and contributes significant insights into neuronal functional differentiation within neural networks.

The MEM-XANES model was trained to predict multiple elements K-edge XANES simultaneously through the innovative combination of a multi-element converged dataset and an elaborated topological approach. This model effectively learns the relationships between the local structures of different absorbing elements and their corresponding XANES spectra, significantly enhancing the prediction accuracy for small-scale sample elements. Remarkably, the MAE achieved by the MEM-XANES model on the EM-Dataset is approximately 0.023, with training time reduced to 0.14 of the original duration due to the absorption atom-centered topological strategy.

Furthermore, the MEM-XANES model demonstrates extraordinary interpretability across three critical dimensions: the neurons of the model, the homology of the data, and topological structures. Notably, a novel perspective is explored that characterizes the behavior of neurons through their response dynamics to different samples for insights into the internal workings of the model. This innovative characterization method revealed the presence of neuronal functional differentiation in the model, guided by the attention mechanism, akin to the functional differentiation observed in cortical areas. In addition, analysis of the homology of the data demonstrates that the model indeed realizes the transformation from crystal structure information to implicit XANES information with multiple semantic separable space *via* graph convolution. In this way, different samples of similar absorbing elements from the same period can mutually learn from each other. The topological structure analysis of the samples indicates the significance of attention scores in distinguishing between atomic environments and reveals a collaborative working pattern between multiple attention heads.

Consequently, this research not only advances future studies on the relationship between XANES spectra and material structures but also provides valuable insights into neuronal behavior, enhancing the understanding of the internal workings of neural networks.

## Author contributions

Chenyu Huang (first author): conceptualization, methodology, software, investigation, formal analysis, writing – original draft; Yunjiang Zhang: formal analysis, supervision; Shuyuan Li: visualization, supervision; Huimin Wang: writing – review & editing; Yaxin Wang: writing – review & editing; Shihao Wei: data curation, validation; Shaorui Sun (corresponding author): conceptualization, funding acquisition, resources, project administration, writing – review & editing.

## Conflicts of interest

There are no conflicts to declare.

## Supplementary Material

SC-016-D5SC00494B-s001

## Data Availability

The code in this publication is publicly available at https://github.com/Shaoruisun/Light-Transition-Metals-K-edge-XANES.
