# Supplementary material for: An explainable “family bucket” model for simultaneous prediction of K-edge XANES for multiple light transition metals
Source: Chem Sci. 2025 Aug 1;16(34):15571–86. doi: 10.1039/d5sc00494b (PMC12315256; doi:10.1039/d5sc00494b)

Supplementary Information (SI) for Chemical Science.
This journal is © The Royal Society of Chemistry 2025

Supporting Information: An Explainable "Family Bucket" Model for Simultaneous Prediction of Multiple Light Transition Metals K-edge XANES

Chenyu Huang[1], Yunjiang Zhang[1], Shuyuan Li[1], Huimin Wang[1], Yaxin Wang[1], Shihao Wei[1], and Shaorui Sun[1,2*]

[1]Department of Chemical Engineering and Technology, College of Materials Science and Engineering, Beijing University of Technology, Beijing 100124, P. R. China; [2]Institute of Matter Science, Beijing University of Technology, Beijing 100124, P. R. China.

Contents

Table S1. Distribution of different elements of data after EM-Dataset splitting. **3**

Table S2~5. Multiple Repeat Experiments Scoring Tables. **4-7**

Figure S1~2. Comparison between calculated and predicted values for samples. **8-9**

Figure S3. Umap dimensionality reduction maps for neurons in layers 1-6.......................... **10**

Figure S4. Layer 4 neurons splitting staining map. **11**

Table S6~11. The source attribution of neurons. **12-17**

Figure S5. Crystal structures of samples in localized zoom of region 2. .... **18**

Table S12~21he sample normalized attention score table. **19-28**

Figure S6. Comparison between FDMNES calculated and predicted values for samples. **29**

Table S1. Distribution of different elements of data after EM-Dataset splitting. The distribution of samples for different absorbing elements before and after EM-Dataset splitting is generally consistent, which provides a strong assurance for training and evaluation by utilizing EM-Dataset model.

Elements	EM-Dataset	Train Dataset	Val Dataset	Test Dataset
Sc	1100	873	106	121
Ti	3756	3000	387	369
V	4203	3343	434	426
Cr	2104	1686	216	202
Mn	5135	4062	538	535
Fe	5038	4020	490	528
Co	4082	3244	406	432
Ni	5081	4077	497	507
Cu	5506	4470	514	522
Zn	5326	4292	548	486
Y	1948	1547	198	203
Zr	1674	1311	180	183
Nb	2264	1817	228	219
Mo	2869	2280	292	297
Tc	124	96	12	16
Ru	785	632	85	68
Rh	864	708	76	80
Pd	1460	1156	131	173
Ag	1803	1476	174	153
Cd	2527	2029	252	246

Table S2. Scoring table for multiple runs on different datasets (EM-Dataset & X-Dataset). Repeating the experiment multiple times reduces the random error and improves the reliability of the experimental result.

Datasets	Test1 R^2	Test2 R^2	Test3 R^2	Test1 MAE	Test2 MAE	Test3 MAE
EM-Dataset	0.9799	0.9794	0.9779	0.0231	0.0233	0.0235
Sc-Dataset	0.9486	0.9546	0.9543	0.0455	0.0428	0.0435
Ti-Dataset	0.9782	0.9772	0.9776	0.0339	0.0345	0.0338
V-Dataset	0.9719	0.9694	0.9719	0.0372	0.0374	0.0375
Cr-Dataset	0.9718	0.9745	0.9729	0.0354	0.0351	0.036
Mn-Dataset	0.9615	0.9561	0.9602	0.0341	0.0338	0.0338
Fe-Dataset	0.9707	0.9695	0.9714	0.0306	0.0305	0.0312
Co-Dataset	0.9534	0.9609	0.9632	0.0375	0.0372	0.0364
Ni-Dataset	0.9824	0.9840	0.9833	0.0252	0.0248	0.0255
Cu-Dataset	0.9783	0.9793	0.9798	0.0252	0.0248	0.0243
Zn-Dataset	0.9713	0.9706	0.9700	0.0229	0.0239	0.0233
Y-Dataset	0.9812	0.9802	0.981	0.0228	0.0237	0.0226
Zr-Dataset	0.9396	0.9559	0.9411	0.0222	0.0231	0.0229
Nb-Dataset	0.9866	0.9856	0.9859	0.0144	0.0153	0.0155
Mo-Dataset	0.9859	0.9861	0.9849	0.016	0.0165	0.0168
Tc-Dataset	0.9301	0.9383	0.9376	0.0555	0.0522	0.0535
Ru-Dataset	0.9887	0.9866	0.9876	0.0194	0.0207	0.0205
Rh-Dataset	0.9895	0.9897	0.9866	0.0164	0.0162	0.0165
Pd-Dataset	0.9867	0.9849	0.9874	0.014	0.0149	0.0134
Ag-Dataset	0.9874	0.988	0.9882	0.0151	0.0138	0.0142
Cd-Dataset	0.9819	0.9814	0.9816	0.0094	0.0102	0.0098

Table S3. Scoring table for multiple runs on different datasets (DIY-Dataset &Tc-Dataset). Repeating the experiment multiple times reduces the random error and improves the reliability of the experimental result.

Test score	DIY-Dataset	Tc-Dataset
Test1 R^2	0.9920	0.9301
Test2 R^2	0.9952	0.9383
Test3 R^2	0.9936	0.9376
Test1 MAE	0.0164	0.0555
Test2 MAE	0.0122	0.0522
Test3 MAE	0.0142	0.0535

Table S4. Scoring table for multiple runs on different models (S GCN: simple graph convolution; xH GAN: x-head graph attention convolution). Repeating the experiment multiple times reduces the random error and improves the reliability of the experimental result.

Test score	S GCN	1H GAN	3H GAN	5H GAN
Test1 R2	0.9675	0.9781	0.9799	0.9786
Test2 R2	0.9678	0.9774	0.9794	0.9795
Test3 R2	0.9664	0.9784	0.9779	0.978
Test1 MAE	0.0322	0.0246	0.0231	0.0234
Test2 MAE	0.0321	0.0247	0.0233	0.0231
Test3 MAE	0.0325	0.0245	0.0235	0.0234

Table S5. Scoring table for multiple runs with different topology approaches (Full Crystal Topology & Atomic environment Topology). Repeating the experiment multiple times reduces the random error and improves the reliability of the experimental result.

Test score	Full Crystal Topology	Atomic environment Topology
Test1 R^2	0.9920	0.9301
Test2 R^2	0.9952	0.9383
Test3 R^2	0.9936	0.9376
Test1 MAE	0.0164	0.0555
Test2 MAE	0.0122	0.0522
Test3 MAE	0.0142	0.0535
Test1 Time Total(s)	45421.5494	5919.3133
Test2 Time Total(s)	46326.3001	5919.3133
Test3 Time Total(s)	46542.5493	7088.6972

Figure S1. The comparison of between the calculated and predicted values for samples in the first quartile(Q1) sorted by MAE from low to high.

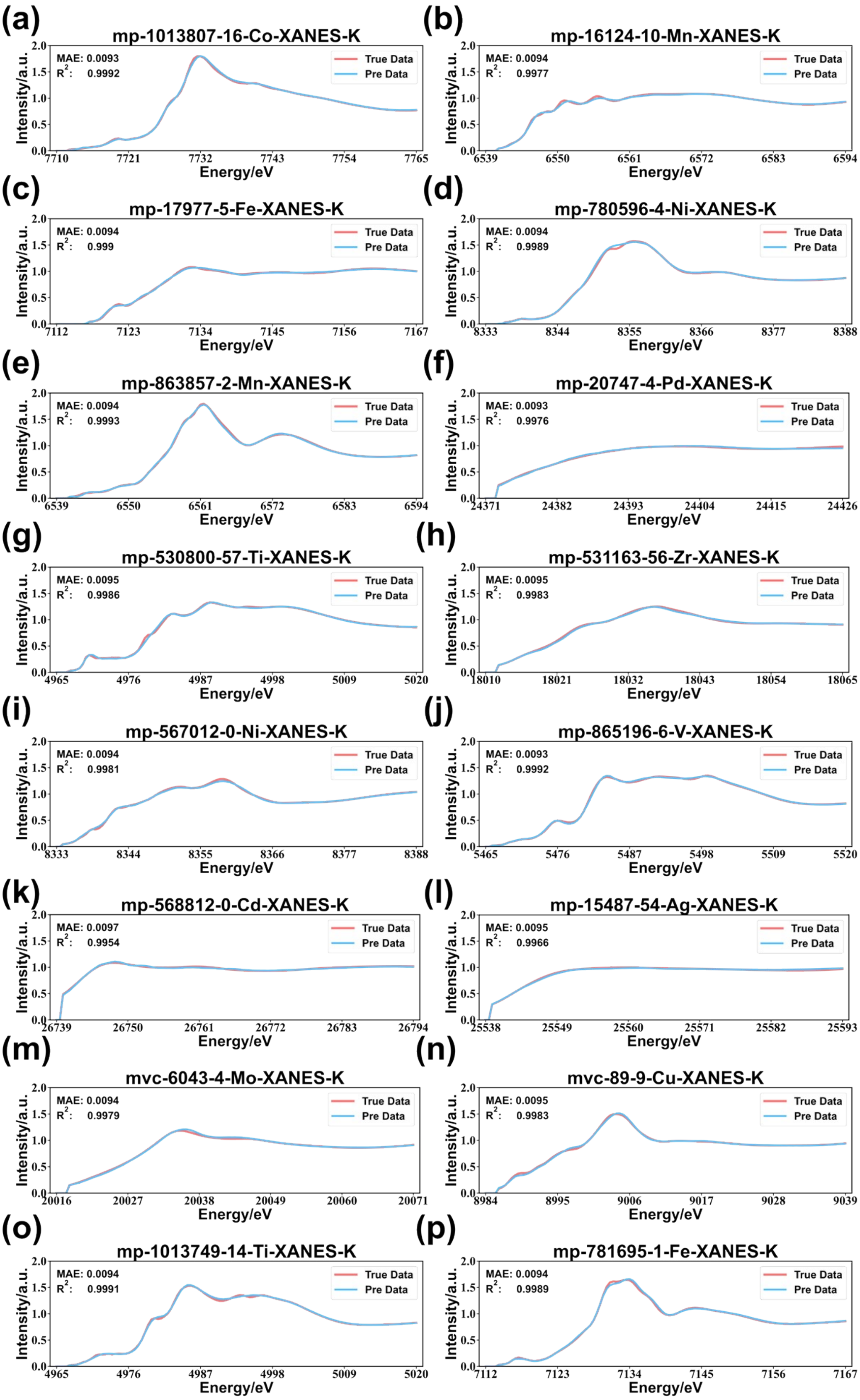

Figure S2. The comparison of between the calculated and predicted values for samples in the third quartile(Q3) sorted by MAE from low to high.

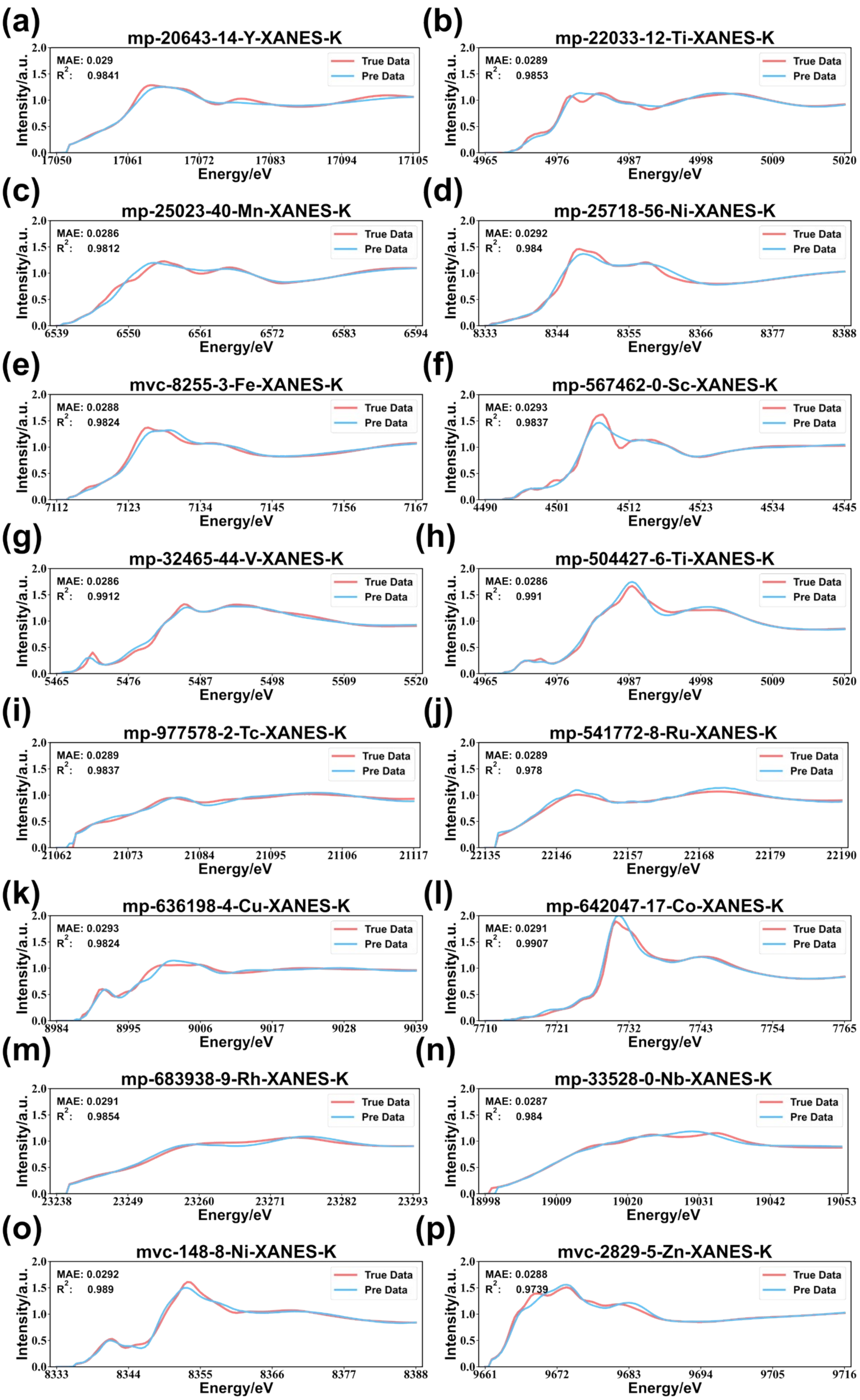

Figure S3. Umap dimensionality reduction maps for neurons in layers 1-6. Only the fourth layer of neurons showed prominent clustering result.

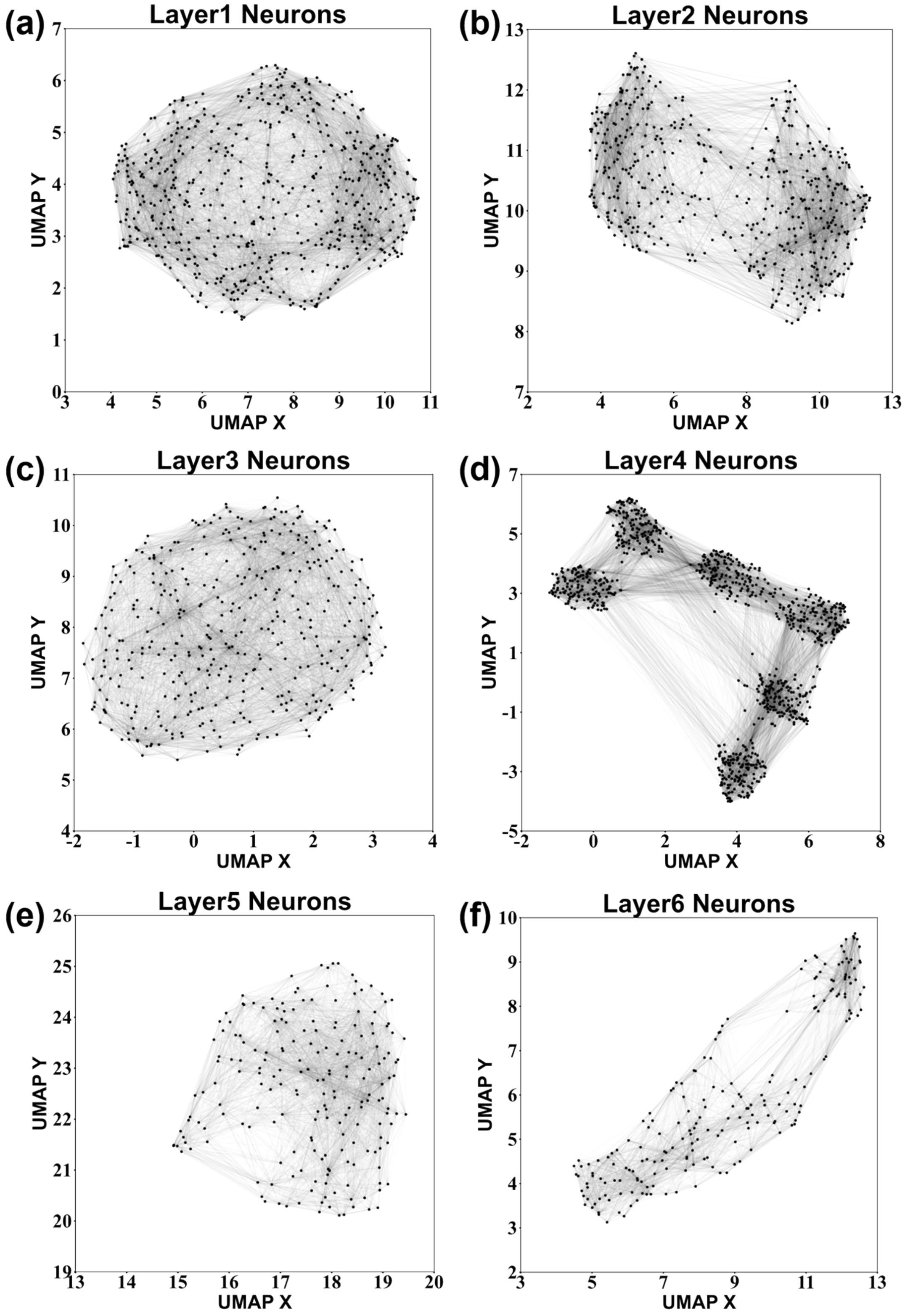

Figure S4. Layer 4 neurons splitting staining map. Straight lines were created to divide the neurons into six regions based on the clustering result.

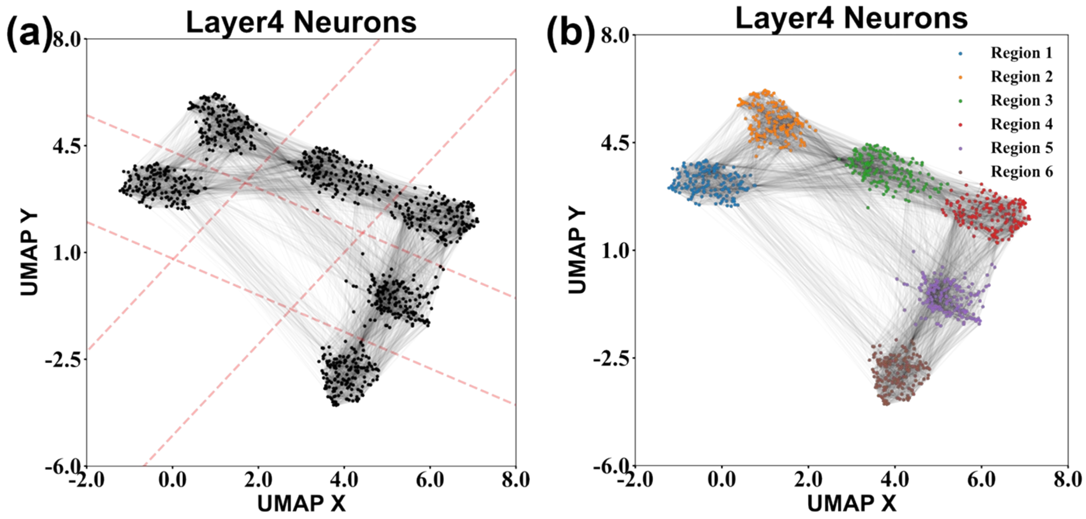

Table S6. The source attribution of neurons.

	Region 1
Rule	The average first ionization energy of near-neighboring atoms is greater than 850 kJ/mol.
Accuracy	85.87%
Neurons	h1-1, h1-2, h1-3, h1-5, h1-6, h1-8, h1-9, h1-13, h1-14, h1-15, h1-20, h1-21, h1-22, h1-24, h1-26, h1-27, h1-31, h1-36, h1-38, h1-39, h1-40, h1-41, h1-46, h1-47, h1-49, h1-55, h1-56, h1-58, h1-59, h1-61, h1-63, h1-65, h1-67, h1-71, h1-76, h1-78, h1-79, h1-80, h1-81, h1-82, h1-83, h1-86, h1-88, h1-90, h1-91, h1-92, h1-93, h1-94, h1-95, h1-96, h1-97, h1-99, h1-100, h1-102, h1-104, h1-106, h1-109, h1-111, h1-113, h1-114, h1-115, h1-116, h1-128, h1-129, h1-130, h1-133, h1-136, h1-139, h1-144, h1-145, h1-151, h1-152, h1-153, h1-154, h1-155, h1-161, h1-162, h1-163, h1-165, h1-168, h1-170, h1-176, h1-178, h1-179, h1-180, h1-182, h1-185, h1-187, h1-188, h1-190, h1-191, h1-194, h1-196, h1-197, h1-198, h1-203, h1-204, h1-206, h1-209, h1-210, h1-211, h1-212, h1-213, h1-215, h1-219, h1-224, h1-225, h1-226, h1-229, h1-232, h1-233, h1-235, h1-237, h1-239, h1-240, h1-241, h1-244, h1-245, h1-251, h1-252, h1-253, h1-254, h1-255, h1-256, h1-258, h1-260, h1-261, h1-264, h1-265, h1-268, h1-269, h1-272, h1-273, h1-274, h1-276, h1-277, h1-278, h1-280, h1-284, h1-286, h1-288, h1-290, h1-292, h1-293, h1-295, h1-296, h1-297, h1-298, h1-299

Table S7. The source attribution of neurons.

	Region 2
Rule	The two nearest neighbor atoms are N, O or F.
Accuracy	97.75%
Neurons	h3-2, h3-3, h3-4, h3-5, h3-12, h3-13, h3-16, h3-18, h3-21, h3-22, h3-25, h3-29, h3-30, h3-31, h3-33, h3-35, h3-37, h3-43, h3-44, h3-45, h3-46, h3-51, h3-52, h3-54, h3-56, h3-58, h3-59, h3-61, h3-62, h3-64, h3-65, h3-66, h3-67, h3-70, h3-71, h3-72, h3-73, h3-74, h3-77, h3-78, h3-80, h3-81, h3-83, h3-84, h3-86, h3-88, h3-90, h3-91, h3-94, h3-95, h3-96, h3-97, h3-98, h3-100, h3-101, h3-103, h3-104, h3-107, h3-109, h3-110, h3-111, h3-112, h3-113, h3-115, h3-118, h3-120, h3-125, h3-128, h3-132, h3-133, h3-134, h3-137, h3-138, h3-140, h3-147, h3-149, h3-151, h3-153, h3-154, h3-160, h3-161, h3-168, h3-170, h3-172, h3-173, h3-176, h3-178, h3-179, h3-180, h3-181, h3-183, h3-184, h3-185, h3-188, h3-189, h3-201, h3-202, h3-203, h3-204, h3-208, h3-209, h3-211, h3-214, h3-218, h3-219, h3-223, h3-224, h3-226, h3-228, h3-235, h3-237, h3-238, h3-239, h3-240, h3-241, h3-242, h3-244, h3-246, h3-251, h3-252, h3-254, h3-255, h3-259, h3-260, h3-261, h3-262, h3-264, h3-265, h3-267, h3-268, h3-270, h3-272, h3-274, h3-275, h3-276, h3-278, h3-280, h3-281, h3-283, h3-284, h3-287, h3-288, h3-289, h3-292, h3-293, h3-294, h3-295, h3-296, h3-297

Table S8. The source attribution of neurons.

	Region 3
Rule	The average first ionization energy of the first shell($\Delta r<0.6$Å) is less than 1300 kJ/mol.
Accuracy	86.63%
Neurons	h2-2, h2-10, h2-11, h2-13, h2-14, h2-17, h2-18, h2-20, h2-22, h2-24, h2-27, h2-28, h2-29, h2-30, h2-31, h2-32, h2-34, h2-37, h2-38, h2-40, h2-41, h2-42, h2-43, h2-45, h2-46, h2-47, h2-52, h2-53, h2-54, h2-59, h2-60, h2-62, h2-64, h2-66, h2-69, h2-70, h2-72, h2-74, h2-78, h2-79, h2-80, h2-85, h2-86, h2-88, h2-89, h2-91, h2-93, h2-94, h2-98, h2-99, h2-100, h2-101, h2-103, h2-105, h2-106, h2-107, h2-111, h2-114, h2-116, h2-118, h2-119, h2-128, h2-130, h2-132, h2-134, h2-138, h2-142, h2-143, h2-145, h2-148, h2-149, h2-150, h2-151, h2-152, h2-154, h2-156, h2-159, h2-160, h2-161, h2-169, h2-171, h2-172, h2-175, h2-177, h2-179, h2-180, h2-181, h2-183, h2-185, h2-186, h2-188, h2-190, h2-191, h2-192, h2-193, h2-194, h2-195, h2-197, h2-200, h2-204, h2-207, h2-209, h2-210, h2-215, h2-217, h2-219, h2-223, h2-225, h2-230, h2-231, h2-232, h2-233, h2-234, h2-236, h2-237, h2-240, h2-242, h2-243, h2-246, h2-248, h2-249, h2-250, h2-253, h2-257, h2-260, h2-261, h2-263, h2-265, h2-267, h2-270, h2-272, h2-274, h2-277, h2-279, h2-282, h2-283, h2-284, h2-285, h2-286, h2-287, h2-289, h2-290, h2-292, h2-293, h2-294, h2-295, h2-296, h2-297, h2-298, h2-299, h3-215

Table S9. The source attribution of neurons.

	Region 4
Rule	The average distance to the first shell($\Delta r<0.6$Å) is less than 2.24Å.
Accuracy	87.60%
Neurons	h2-1, h2-3, h2-4, h2-5, h2-6, h2-7, h2-8, h2-9, h2-12, h2-15, h2-16, h2-19, h2-21, h2-23, h2-25, h2-26, h2-33, h2-35, h2-36, h2-39, h2-44, h2-48, h2-49, h2-50, h2-51, h2-55, h2-56, h2-57, h2-58, h2-61, h2-63, h2-65, h2-67, h2-68, h2-71, h2-73, h2-75, h2-76, h2-77, h2-81, h2-82, h2-83, h2-84, h2-87, h2-90, h2-92, h2-95, h2-96, h2-97, h2-102, h2-104, h2-108, h2-109, h2-110, h2-112, h2-113, h2-115, h2-117, h2-120, h2-122, h2-123, h2-124, h2-125, h2-126, h2-127, h2-129, h2-131, h2-133, h2-135, h2-136, h2-137, h2-139, h2-140, h2-141, h2-144, h2-146, h2-147, h2-153, h2-155, h2-157, h2-158, h2-162, h2-163, h2-164, h2-165, h2-166, h2-167, h2-168, h2-170, h2-173, h2-174, h2-176, h2-178, h2-182, h2-184, h2-187, h2-189, h2-196, h2-198, h2-199, h2-201, h2-202, h2-203, h2-205, h2-206, h2-208, h2-211, h2-212, h2-213, h2-214, h2-216, h2-218, h2-220, h2-221, h2-222, h2-224, h2-226, h2-227, h2-228, h2-229, h2-235, h2-238, h2-239, h2-241, h2-244, h2-245, h2-247, h2-251, h2-252, h2-254, h2-255, h2-256, h2-258, h2-259, h2-262, h2-264, h2-266, h2-268, h2-269, h2-271, h2-273, h2-275, h2-276, h2-278, h2-280, h2-281, h2-288, h2-291, h2-300

Table S10. The source attribution of neurons.

	Region 5
Rule	The nearest neighboring atom distance is less than 2.20Å.
Accuracy	92.23%
Neurons	h3-1, h3-6, h3-7, h3-8, h3-9, h3-10, h3-11, h3-14, h3-15, h3-17, h3-19, h3-20, h3-23, h3-24, h3-26, h3-27, h3-28, h3-32, h3-34, h3-36, h3-38, h3-39, h3-40, h3-41, h3-42, h3-47, h3-48, h3-49, h3-50, h3-53, h3-55, h3-57, h3-60, h3-63, h3-68, h3-69, h3-75, h3-76, h3-79, h3-82, h3-85, h3-87, h3-89, h3-92, h3-93, h3-99, h3-102, h3-105, h3-106, h3-108, h3-114, h3-116, h3-117, h3-119, h3-121, h3-122, h3-123, h3-124, h3-126, h3-127, h3-129, h3-130, h3-131, h3-135, h3-136, h3-139, h3-141, h3-142, h3-143, h3-144, h3-145, h3-146, h3-148, h3-150, h3-152, h3-155, h3-156, h3-157, h3-158, h3-159, h3-162, h3-163, h3-164, h3-165, h3-166, h3-167, h3-169, h3-171, h3-174, h3-175, h3-177, h3-182, h3-186, h3-187, h3-190, h3-191, h3-192, h3-193, h3-194, h3-195, h3-196, h3-197, h3-198, h3-199, h3-200, h3-205, h3-206, h3-207, h3-210, h3-212, h3-213, h3-216, h3-217, h3-220, h3-221, h3-222, h3-225, h3-227, h3-229, h3-230, h3-231, h3-232, h3-233, h3-234, h3-236, h3-243, h3-245, h3-247, h3-248, h3-249, h3-250, h3-253, h3-256, h3-257, h3-258, h3-263, h3-266, h3-269, h3-271, h3-273, h3-277, h3-279, h3-282, h3-285, h3-286, h3-290, h3-291, h3-298, h3-299, h3-300

Table S11. The source attribution of neurons.

Region 6	
Rule	The average distance of neighbor atoms within 2.70Å is less than 2.30Å.
Accuracy	93.13%
Neurons	h1-4, h1-7, h1-10, h1-11, h1-12, h1-16, h1-17, h1-18, h1-19, h1-23, h1-25, h1-28, h1-29, h1-30, h1-32, h1-33, h1-34, h1-35, h1-37, h1-42, h1-43, h1-44, h1-45, h1-48, h1-50, h1-51, h1-52, h1-53, h1-54, h1-57, h1-60, h1-62, h1-64, h1-66, h1-68, h1-69, h1-70, h1-72, h1-73, h1-74, h1-75, h1-77, h1-84, h1-85, h1-87, h1-89, h1-98, h1-101, h1-103, h1-105, h1-107, h1-108, h1-110, h1-112, h1-117, h1-118, h1-119, h1-120, h1-121, h1-122, h1-123, h1-124, h1-125, h1-126, h1-127, h1-131, h1-132, h1-134, h1-135, h1-137, h1-138, h1-140, h1-141, h1-142, h1-143, h1-146, h1-147, h1-148, h1-149, h1-150, h1-156, h1-157, h1-158, h1-159, h1-160, h1-164, h1-166, h1-167, h1-169, h1-171, h1-172, h1-173, h1-174, h1-175, h1-177, h1-181, h1-183, h1-184, h1-186, h1-189, h1-192, h1-193, h1-195, h1-199, h1-200, h1-201, h1-202, h1-205, h1-207, h1-208, h1-214, h1-216, h1-217, h1-218, h1-220, h1-221, h1-222, h1-223, h1-227, h1-228, h1-230, h1-231, h1-234, h1-236, h1-238, h1-242, h1-243, h1-246, h1-247, h1-248, h1-249, h1-250, h1-257, h1-259, h1-262, h1-263, h1-266, h1-267, h1-270, h1-271, h1-275, h1-279, h1-281, h1-282, h1-283, h1-285, h1-287, h1-289, h1-291, h1-294, h1-300, h2-121

Figure S5. Crystal structures of samples in localized zoom of region 2. The more closely spaced samples in the T-SNE are indeed from extremely similar localizations.

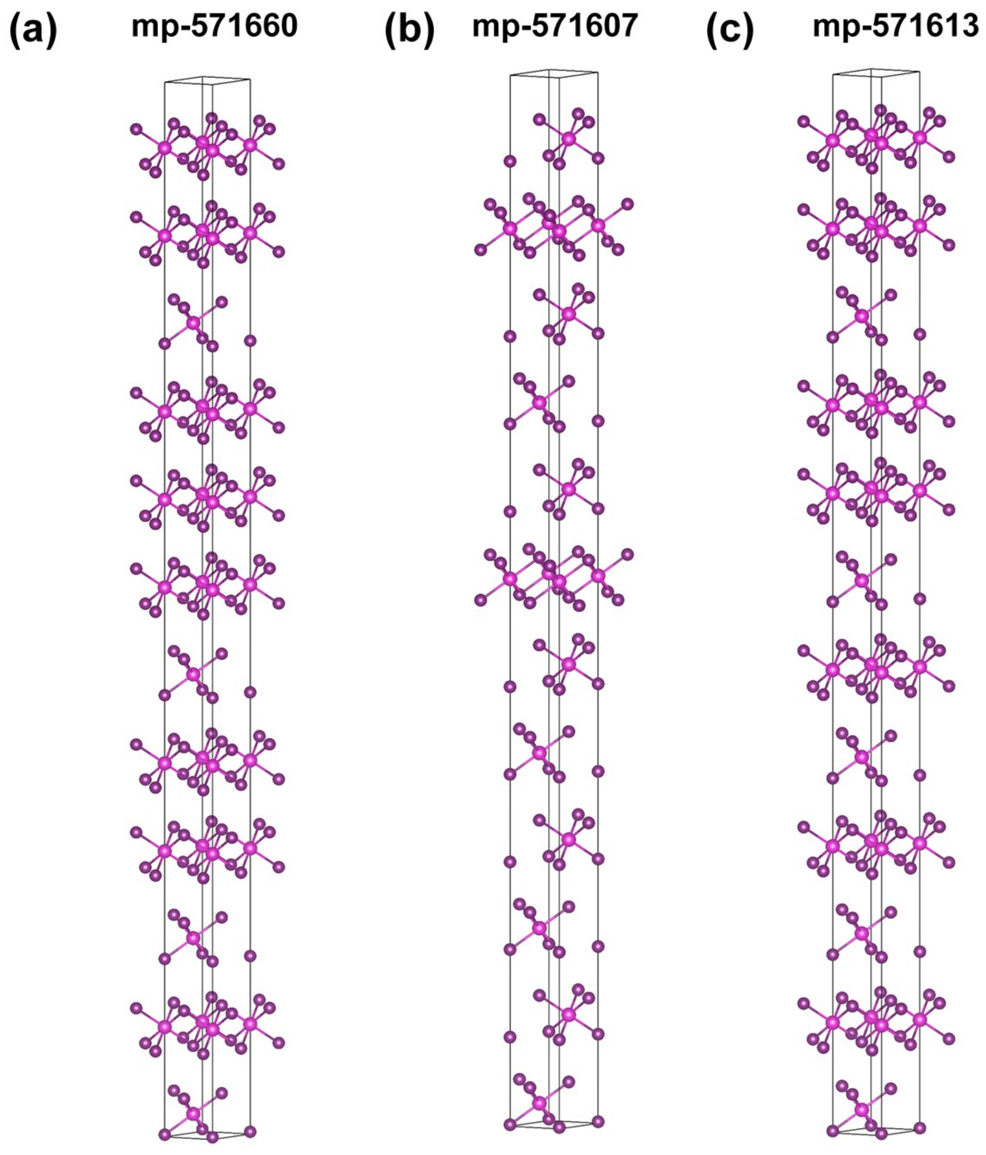

Table S12. The sample mp-1005986-26-Mo-XANES-K (absence) normalized attention score table.

ID	Neighbor atom Symbol	Distance(Å)	Head1 Δscore	Head2 Δscore	Head3 Δscore
21	Mo	2.701	4.819E-01	9.976E-01	1.649E-01
20	Mo	2.701	4.819E-01	9.976E-01	1.649E-01
27	Mo	2.918	4.650E-01	1.000E+00	1.632E-01
23	Mo	2.918	4.650E-01	1.000E+00	1.632E-01
16	Zr	2.943	8.396E-01	5.835E-01	1.217E-03
9	Zr	2.943	8.396E-01	5.835E-01	1.217E-03
1	Zr	2.951	8.399E-01	5.835E-01	1.151E-03
5	Zr	2.951	8.399E-01	5.835E-01	1.151E-03
11	Zr	3.116	8.537E-01	5.830E-01	0.000E+00
14	Zr	3.116	8.537E-01	5.830E-01	0.000E+00
7	Zr	3.116	8.537E-01	5.830E-01	0.000E+00
12	Zr	3.116	8.537E-01	5.830E-01	0.000E+00
19	Co	4.487	0.000E+00	0.000E+00	1.000E+00
19	Co	4.487	0.000E+00	0.000E+00	1.000E+00
22	Mo	4.546	6.001E-01	9.892E-01	1.618E-01
22	Mo	4.546	6.001E-01	9.892E-01	1.618E-01
24	Mo	4.546	6.001E-01	9.892E-01	1.618E-01
24	Mo	4.546	6.001E-01	9.892E-01	1.618E-01
17	Zr	4.915	1.000E+00	5.773E-01	1.077E-03
6	Zr	4.915	1.000E+00	5.773E-01	1.077E-03

Table S13. The sample mvc-16233-0-Zr-XANES-K (absence) normalized attention score table.

Neighbor atom			Head1 Δscore	Head2 Δscore	Head3 Δscore
ID	Symbol	Distance(Å)			
11	Zn	2.756	9.919E-01	3.853E-03	1.000E+00
10	Zn	2.756	9.919E-01	3.853E-03	1.000E+00
15	Sb	2.966	0.000E+00	3.272E-01	2.798E-03
12	Sb	2.966	0.000E+00	3.272E-01	2.798E-03
16	Sb	2.996	1.544E-04	3.274E-01	2.208E-03
14	Sb	3.111	6.391E-04	3.283E-01	0.000E+00
14	Sb	3.111	6.391E-04	3.283E-01	0.000E+00
7	Zr	3.526	9.330E-01	9.968E-01	4.008E-01
6	Zr	3.526	9.330E-01	9.968E-01	4.008E-01
9	Zr	3.526	9.330E-01	9.968E-01	4.008E-01
8	Zr	3.526	9.330E-01	9.968E-01	4.008E-01
1	Zr	3.754	9.345E-01	9.991E-01	4.004E-01
1	Zr	3.754	9.345E-01	9.991E-01	4.004E-01
4	Zr	3.754	9.345E-01	9.991E-01	4.004E-01
4	Zr	3.754	9.345E-01	9.991E-01	4.004E-01
3	Zr	4.037	9.355E-01	1.000E+00	4.001E-01
2	Zr	4.037	9.355E-01	1.000E+00	4.001E-01
5	Zr	4.970	9.335E-01	9.777E-01	3.964E-01
5	Zr	4.970	9.335E-01	9.777E-01	3.964E-01
11	Zn	4.993	1.000E+00	0.000E+00	9.880E-01

Table S14. The sample mp-780940-13-Fe-XANES-K (absence) normalized attention score table.

	Neighbor atom				
ID	Symbol	Distance(Å)	Head1 Δscore	Head2 Δscore	Head3 Δscore
43	O	2.034	2.133E-01	1.664E-02	4.843E-01
42	O	2.053	2.192E-01	1.638E-02	4.859E-01
39	O	2.073	2.256E-01	1.622E-02	4.877E-01
34	O	2.165	2.567E-01	1.682E-02	4.971E-01
46	O	2.436	3.204E-01	2.530E-02	5.255E-01
7	Li	2.941	7.147E-01	1.258E-03	5.953E-05
19	B	2.983	4.159E-03	1.000E+00	7.527E-01
6	Li	2.988	7.132E-01	1.044E-03	2.101E-05
18	B	2.993	3.963E-03	9.992E-01	7.524E-01
21	B	3.011	3.629E-03	9.980E-01	7.520E-01
21	B	3.065	2.576E-03	9.943E-01	7.506E-01
4	Li	3.072	7.109E-01	6.963E-04	0.000E+00
6	Li	3.101	7.102E-01	5.840E-04	7.003E-06
12	Fe	3.129	1.000E+00	9.056E-02	1.000E+00
33	O	3.194	3.187E-01	2.728E-02	5.135E-01
23	B	3.212	0.000E+00	9.861E-01	7.472E-01
15	Fe	3.256	9.972E-01	8.974E-02	9.984E-01
37	O	3.277	3.162E-01	2.690E-02	5.117E-01
42	O	3.314	3.151E-01	2.674E-02	5.110E-01
3	Li	3.409	7.058E-01	0.000E+00	7.353E-04

Table S15. The sample mp-10117-19-Ni-XANES-K (absence) normalized attention score table.

ID	Neighbor atom Symbol	Distance(Å)	Head1 Δscore	Head2 Δscore	Head3 Δscore
0	B	2.088	0.000E+00	8.941E-01	3.217E-01
2	B	2.088	0.000E+00	8.941E-01	3.217E-01
16	Ni	2.384	9.891E-01	0.000E+00	1.305E-01
22	Ni	2.540	9.986E-01	1.567E-03	1.267E-01
13	Ni	2.540	9.986E-01	1.567E-03	1.267E-01
8	Mg	2.540	3.555E-01	4.591E-01	1.000E+00
21	Ni	2.540	9.986E-01	1.567E-03	1.267E-01
15	Ni	2.540	9.986E-01	1.567E-03	1.267E-01
26	Ni	2.640	1.000E+00	2.441E-03	1.115E-01
25	Ni	2.640	1.000E+00	2.441E-03	1.115E-01
14	Ni	2.640	1.000E+00	2.441E-03	1.115E-01
23	Ni	2.640	1.000E+00	2.441E-03	1.115E-01
7	Mg	2.896	3.536E-01	4.843E-01	9.410E-01
6	Mg	2.896	3.536E-01	4.843E-01	9.410E-01
1	B	3.537	2.038E-02	9.977E-01	2.963E-01
5	B	3.537	2.038E-02	9.977E-01	2.963E-01
17	Ni	3.593	9.777E-01	3.978E-03	0.000E+00
20	Ni	3.593	9.777E-01	3.978E-03	0.000E+00
4	B	3.826	1.986E-02	1.000E+00	2.930E-01
3	B	3.826	1.986E-02	1.000E+00	2.930E-01

Table S16. The sample mp-10138-23-Ru-XANES-K (absence) normalized attention score table.

ID	Neighbor atom Symbol	Distance(Å)	Head1 Δscore	Head2 Δscore	Head3 Δscore
15	B	2.148	7.191E-01	0.000E+00	1.464E-01
5	B	2.172	7.217E-01	2.315E-04	1.497E-01
8	B	2.219	7.258E-01	7.740E-04	1.553E-01
3	B	2.288	7.294E-01	1.692E-03	1.612E-01
7	B	2.292	7.296E-01	1.745E-03	1.615E-01
30	Ru	2.725	9.318E-03	9.846E-01	1.000E+00
29	Ru	2.725	9.318E-03	9.846E-01	1.000E+00
27	Ru	2.773	8.129E-03	9.864E-01	9.958E-01
24	Ru	2.815	7.150E-03	9.877E-01	9.923E-01
18	Y	2.929	1.000E+00	6.699E-01	4.573E-03
31	Ru	2.994	3.708E-03	9.915E-01	9.799E-01
25	Ru	3.123	1.981E-03	9.934E-01	9.740E-01
12	B	3.145	7.187E-01	5.585E-03	1.317E-01
16	Y	3.165	9.982E-01	6.685E-01	5.121E-04
17	Y	3.226	9.979E-01	6.682E-01	0.000E+00
11	B	3.720	7.189E-01	5.578E-03	1.300E-01
6	B	3.737	7.190E-01	5.585E-03	1.302E-01
2	B	3.737	7.190E-01	5.585E-03	1.302E-01
35	Ru	4.049	0.000E+00	1.000E+00	9.714E-01
35	Ru	4.049	0.000E+00	1.000E+00	9.714E-01

Table S17. The sample mp-640381-6-Cu-XANES-K (presence) normalized attention score table.

Neighbor atom			Head1 Δscore	Head2 Δscore	Head3 Δscore
ID	Symbol	Distance(Å)			
13	S	2.220	4.650E-02	1.640E-01	5.106E-03
12	S	2.321	4.616E-02	1.636E-01	4.680E-03
10	S	2.348	4.612E-02	1.636E-01	4.691E-03
10	S	2.359	4.611E-02	1.636E-01	4.709E-03
4	Cu	2.966	0.000E+00	0.000E+00	0.000E+00
4	Cu	2.966	2.229E-07	0.000E+00	0.000E+00
2	Yb	3.318	2.414E-02	1.628E-01	1.405E-01
2	Yb	3.322	2.414E-02	1.628E-01	1.405E-01
0	Yb	3.382	2.423E-02	1.628E-01	1.407E-01
12	S	3.921	4.898E-02	1.675E-01	1.206E-02
6	Cu	3.925	1.000E+00	1.000E+00	1.000E+00
6	Cu	3.925	1.000E+00	1.000E+00	1.000E+00
3	Yb	4.422	2.463E-02	1.623E-01	1.431E-01
2	Yb	4.439	2.462E-02	1.623E-01	1.431E-01
2	Yb	4.441	2.462E-02	1.623E-01	1.431E-01
13	S	4.509	4.894E-02	1.665E-01	1.278E-02
13	S	4.509	4.894E-02	1.665E-01	1.278E-02
14	S	4.554	4.892E-02	1.663E-01	1.279E-02
12	S	4.554	4.892E-02	1.663E-01	1.279E-02
12	S	4.565	4.892E-02	1.663E-01	1.279E-02

Table S18. The sample mp-1005829-13-Mn-XANES-K (presence) normalized attention score table.

Neighbor atom			Head1 Δscore	Head2 Δscore	Head3 Δscore
ID	Symbol	Distance(Å)			
15	P	2.422	1.722E-02	9.462E-04	4.101E-03
20	P	2.504	1.680E-02	9.554E-04	3.895E-03
20	P	2.504	1.680E-02	9.554E-04	3.895E-03
17	P	2.556	1.658E-02	9.599E-04	3.591E-03
17	P	2.556	1.658E-02	9.599E-04	3.591E-03
10	Mn	2.695	2.302E-02	0.000E+00	9.352E-02
7	Mn	2.711	2.302E-02	2.758E-07	9.335E-02
7	Mn	2.711	2.302E-02	2.758E-07	9.335E-02
6	Mn	2.767	2.305E-02	1.034E-06	9.277E-02
6	Mn	2.767	2.305E-02	1.034E-06	9.277E-02
4	Mn	2.841	2.310E-02	1.862E-06	9.205E-02
4	Mn	2.841	2.310E-02	1.862E-06	9.205E-02
11	Mn	3.117	2.337E-02	2.827E-06	9.020E-02
12	Mn	3.126	2.338E-02	2.827E-06	9.017E-02
16	P	3.400	1.641E-02	9.638E-04	0.000E+00
0	Sm	3.579	0.000E+00	4.959E-04	2.407E-02
13	Mn	3.630	1.000E+00	1.000E+00	1.000E+00
13	Mn	3.630	1.000E+00	1.000E+00	1.000E+00
9	Mn	4.029	2.383E-02	2.138E-06	9.029E-02
8	Mn	4.049	2.383E-02	2.069E-06	9.031E-02

Table S19. The sample mp-662804-31-Pd-XANES-K (presence) normalized attention score table.

ID	Neighbor atom Symbol	Distance(Å)	Head1 Δscore	Head2 Δscore	Head3 Δscore
14	Sm	2.868	0.000E+00	0.000E+00	9.907E-01
16	Sm	2.869	1.139E-04	7.086E-06	9.907E-01
39	Pd	2.925	9.906E-01	9.843E-01	3.511E-03
35	Pd	2.925	9.906E-01	9.843E-01	3.511E-03
57	Pd	2.928	9.907E-01	9.843E-01	3.376E-03
44	Pd	2.938	9.909E-01	9.845E-01	3.012E-03
13	Sm	2.943	6.076E-03	4.075E-04	9.893E-01
13	Sm	2.944	6.106E-03	4.075E-04	9.893E-01
49	Pd	3.044	9.934E-01	9.866E-01	1.823E-04
56	Pd	3.048	9.935E-01	9.867E-01	1.013E-04
51	Pd	3.054	9.936E-01	9.869E-01	0.000E+00
40	Pd	4.351	9.999E-01	1.000E+00	1.058E-02
40	Pd	4.357	9.999E-01	1.000E+00	1.060E-02
26	Pd	4.397	9.999E-01	9.999E-01	1.076E-02
26	Pd	4.398	9.999E-01	9.999E-01	1.076E-02
42	Pd	4.415	1.000E+00	9.999E-01	1.082E-02
3	Sm	4.459	2.841E-02	2.381E-03	9.942E-01
3	Sm	4.468	2.842E-02	2.367E-03	9.943E-01
50	Pd	4.580	1.000E+00	9.994E-01	1.116E-02
12	Sm	4.836	2.942E-02	1.570E-03	1.000E+00

Table S20. The sample mp-553916-23-Zn-XANES-K (presence) normalized attention score table.

Neighbor atom			Head1 Δscore	Head2 Δscore	Head3 Δscore
ID	Symbol	Distance(Å)			
42	S	2.359	6.826E-04	4.723E-02	3.945E-03
34	S	2.360	6.731E-04	4.723E-02	3.941E-03
34	S	2.360	6.731E-04	4.723E-02	3.941E-03
34	S	2.360	6.731E-04	4.723E-02	3.941E-03
23	Zn	3.853	1.000E+00	1.000E+00	1.000E+00
23	Zn	3.853	1.000E+00	1.000E+00	1.000E+00
23	Zn	3.853	1.000E+00	1.000E+00	1.000E+00
23	Zn	3.853	1.000E+00	1.000E+00	1.000E+00
23	Zn	3.853	1.000E+00	1.000E+00	1.000E+00
23	Zn	3.853	1.000E+00	1.000E+00	1.000E+00
8	Zn	3.856	3.835E-01	0.000E+00	9.170E-02
8	Zn	3.856	3.835E-01	0.000E+00	9.170E-02
8	Zn	3.856	3.835E-01	0.000E+00	9.170E-02
2	Zn	3.856	3.835E-01	0.000E+00	9.170E-02
2	Zn	3.856	3.835E-01	0.000E+00	9.170E-02
2	Zn	3.856	3.835E-01	0.000E+00	9.170E-02
42	S	4.518	0.000E+00	4.820E-02	0.000E+00
42	S	4.518	0.000E+00	4.820E-02	0.000E+00
42	S	4.518	0.000E+00	4.820E-02	0.000E+00
42	S	4.518	0.000E+00	4.820E-02	0.000E+00

Table S21. The sample mp-571605-6-Cd-XANES-K (presence) normalized attention score table.

Neighbor atom			Head1 Δscore	Head2 Δscore	Head3 Δscore
ID	Symbol	Distance(Å)			
25	I	3.039	1.720E-03	7.378E-03	2.390E-03
25	I	3.039	1.720E-03	7.378E-03	2.390E-03
25	I	3.039	1.720E-03	7.378E-03	2.390E-03
21	I	3.039	1.720E-03	7.373E-03	2.390E-03
21	I	3.039	1.720E-03	7.373E-03	2.390E-03
21	I	3.039	1.720E-03	7.373E-03	2.390E-03
6	Cd	4.332	1.000E+00	1.000E+00	1.000E+00
6	Cd	4.332	1.000E+00	1.000E+00	1.000E+00
6	Cd	4.332	1.000E+00	1.000E+00	1.000E+00
6	Cd	4.332	1.000E+00	1.000E+00	1.000E+00
6	Cd	4.332	1.000E+00	1.000E+00	1.000E+00
6	Cd	4.332	1.000E+00	1.000E+00	1.000E+00
25	I	5.291	1.298E-03	0.000E+00	1.559E-03
25	I	5.291	1.298E-03	2.474E-06	1.559E-03
25	I	5.291	1.298E-03	0.000E+00	1.559E-03
21	I	5.291	1.298E-03	2.474E-06	1.559E-03
21	I	5.291	1.298E-03	2.474E-06	1.559E-03
21	I	5.291	1.298E-03	0.000E+00	1.559E-03
11	I	6.235	0.000E+00	6.017E-02	0.000E+00
11	I	6.235	0.000E+00	6.017E-02	0.000E+00

Figure S6. The comparison of between the FDMNES calculated and predicted values for samples.

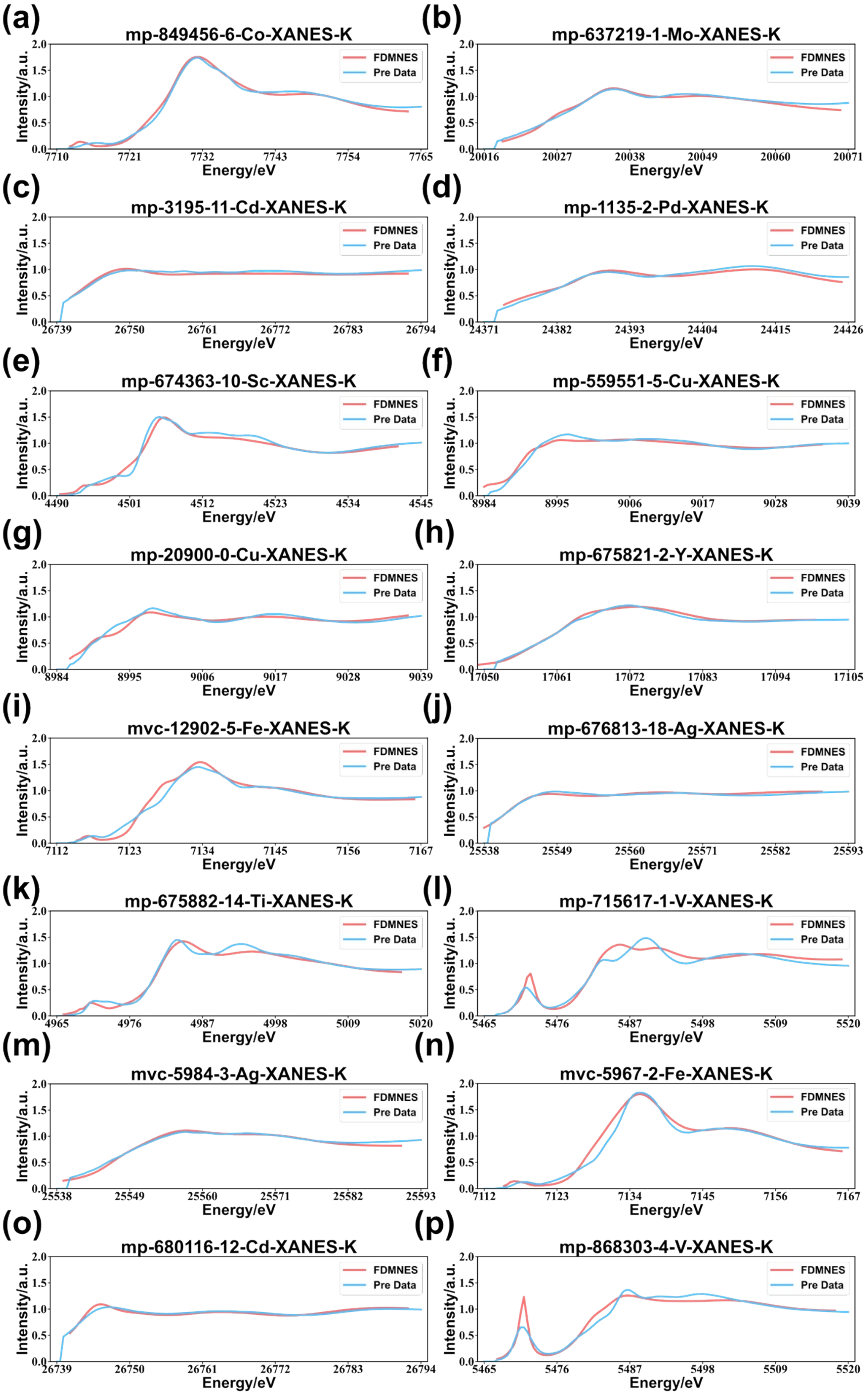

Supplement: SC-016-D5SC00494B-s001 [file SC-016-D5SC00494B-s001.pdf]
